# Circulating Extracellular Vesicles as Putative Mediators of Cardiovascular Disease in Paediatric Chronic Kidney Disease

**DOI:** 10.1002/jev2.70062

**Published:** 2025-03-21

**Authors:** Felix Behrens, Johannes Holle, Chia‐Yu Chen, Laura F. Ginsbach, Benjamin C. Krause, Ulrike Bruning, Fabian L. Kriegel, Toralf Kaiser, István A. Szijártó, Harithaa Anandakumar, Katrin Lehmann, Fabian Schumacher, Pawel Durek, Frederik F. Heinrich, Dörte Lodka, Carina Hoffmann, André A. Borchardt, Lisa Peters, Laura Michalick, Uwe Querfeld, Philip Bufler, Andreas Luch, Burkhard Kleuser, Jennifer A. Kirwan, Sofia K. Forslund, Julia Thumfahrt, Dominik Müller, Nicola Wilck, Mir‐Farzin Mashreghi, Ulrike Löber, Hendrik Bartolomaeus, Wolfgang M. Kuebler, Szandor Simmons

**Affiliations:** ^1^ Institute of Physiology Charité – Universitätsmedizin Berlin Berlin Germany; ^2^ Department of Pediatric Gastroenterology, Nephrology and Metabolic Diseases Charité – Universitätsmedizin Berlin Berlin Germany; ^3^ Berlin Institute of Health (BIH) at Charité – Universitätsmedizin Berlin Berlin Germany; ^4^ DZHK (German Centre for Cardiovascular Research), Partner Site Berlin Berlin Germany; ^5^ Experimental and Clinical Research Center A Cooperation of Charité – Universitätsmedizin Berlin and Max Delbrück Center for Molecular Medicine Berlin Germany; ^6^ Max Delbrück Center for Molecular Medicine in the Helmholtz Association Berlin Germany; ^7^ Department of Chemical and Product Safety German Federal Institute for Risk Assessment (BfR) Berlin Germany; ^8^ Carl Zeiss Meditech Berlin Germany; ^9^ German Rheumatism Research Centre (DRFZ) Berlin Germany; ^10^ Department of Nephrology und Medical Intensive Care Charité – Universitätsmedizin Berlin Berlin Germany; ^11^ Institute of Pharmacy Freie Universität Berlin Berlin Germany; ^12^ Institute of Biology Freie Universität Berlin Berlin Germany; ^13^ Departments of Physiology and Surgery University of Toronto Toronto ON Canada; ^14^ Keenan Research Centre for Biomedical Science St. Michael's Hospital Toronto ON Canada

**Keywords:** chronic kidney disease, cardiovascular disease, microRNAs, angiogenesis, uremic toxins, shear stress

## Abstract

Cardiovascular disease (CVD) is the leading cause of mortality in chronic kidney disease (CKD). However, the pathogenesis of CVD in CKD remains incompletely understood. Endothelial extracellular vesicles (EC‐EVs) have previously been associated with CVD. We hypothesized that CKD alters EV release and cargo, subsequently promoting vascular remodelling. We recruited 94 children with CKD, including patients after kidney transplantation and healthy donors, and performed EV phenotyping and functional EV analyses in the absence of age‐related comorbidities. Plasma EC‐EVs were increased in haemodialysis patients and decreased after kidney transplantation. Thirty microRNAs were less abundant in total CKD plasma EVs with predicted importance in angiogenesis and smooth muscle cell proliferation. In vitro, CKD plasma EVs induced transcriptomic changes in angiogenesis pathways and functionally impaired angiogenic properties, migration and proliferation in ECs. High shear stress, as generated by arterio‐venous fistulas, and uremic toxins were considered as potential drivers of EV release, but only the combination increased EV generation from venous ECs. The resulting EVs recapitulated miRNA changes observed in CKD in vivo. In conclusion, CKD results in the release of EVs with altered miRNA profiles and anti‐angiogenic properties, which may mediate vascular pathology in children with CKD. EVs and their miRNA cargo may represent future therapeutic targets to attenuate CVD in CKD.

## 1 Background

Chronic kidney disease (CKD) is one of the most common non‐communicable diseases worldwide and embodies a global health problem, affecting 8%–16% of the world's population and accounting for 35.8 million disability‐adjusted life years (Jha et al. 2013; GBD Chronic Kidney Disease Collaboration 2020). Cardiovascular disease (CVD) is a common comorbidity in patients with CKD and cardiovascular events form the leading cause of death in these patients, with cardiovascular mortality 10–30 times higher in end‐stage CKD than in the general population (Jha et al. 2013; Gansevoort et al. 2013). Despite ongoing efforts, the mechanisms underlying the development of CVD in CKD remain incompletely understood.

Circulating endothelial cell‐derived (EC) EVs have previously been associated with CVD and CKD (Behrens et al. 2020). Several studies have shown increased levels of EC‐EVs in adult CKD patients (Cavallari et al. 2019; Faure et al. 2006; Burton et al. 2013; Trappenburg et al. 2012; Ariza et al. 2013; Merino et al. 2010; Carmona et al. 2017; Soriano et al. 2014; Amabile et al. 2005; Esquivias‐Motta et al. 2017) who suffer from high cardiovascular morbidity, presumably caused by CKD and/or by common risk factors for CVD and CKD, that is, hypertension and diabetes (Matsushita et al. 2022). Strikingly, CKD patients with extensive CVD have higher EC‐EV concentrations than CKD patients with less pronounced CVD (Chen et al. 2015), suggesting a potential role for EC‐EVs as biomarkers and mediators of CVD in CKD. Children with CKD suffer from a similar increase in cardiovascular morbidity, but rarely have pre‐existing cardiovascular risk factors (Mitsnefes 2012). Therefore, children with CKD represent a uniquely valuable study population to elucidate of kidney‐specific mechanisms of CVD development in CKD.

We hypothesized that CKD drives endothelial EV release and alters EV cargo in affected children, which in turn promotes CVD in CKD in the absence of confounding comorbidities. We provide in‐depth EV phenotyping of a cohort of children at different CKD stages cross‐sectionally compared to age‐matched healthy donors and patients with restored excretory function after kidney transplantation (KTx), and evaluate the longitudinal effects of KTx on EV characteristics. Using in silico and in vitro methods, we further define the role of CKD EVs in CVD and elucidate the mechanisms of EV release in CKD.

## 2 Materials and Methods

Detailed methods and information on statistical analyses are provided in the Supplemental material.

### 2.1 Patient Recruitment

Pediatric CKD patients aged 2–18 years and age‐matched healthy donors were recruited at the Department of Pediatric Gastroenterology, Nephrology and Metabolic Diseases at Charité—Universitätsmedizin Berlin. Patients with acute infections or acute kidney diagnoses were excluded. Informed written consent was obtained from legal guardians and older children/adolescents, as appropriate, in accordance with the Declaration of Helsinki and approval by the Ethics Committee of Charité—Universitätsmedizin Berlin (EA2/162/17). CKD patients were divided into four groups: CKD G3‐G5 patients without dialysis, CKD G5 patients on peritoneal dialysis (PD), CKD G5 patients on haemodialysis or hemodiafiltration (both referred to as HD hereafter) and CKD patients with kidney transplant (KTx) and stable graft function. When available, patients in the CKD, HD or PD groups were followed longitudinally after KTx (>1 month after KTx). Patients with signs of graft rejection, eGFR < 60 mL/min/1.73 m2, worsening eGFR or acute infections were excluded.

Adult haemodialysis patients were recruited to analyse the immediate effects of HD on EC‐EV release. CKD patients receiving maintenance HD were evaluated at the Department of Nephrology and Medical Intensive Care at Charité—Universitätsmedizin Berlin. Patients with acute infections, antibiotic therapy or acute eGFR decline were excluded. Informed written consent was obtained in accordance with the Declaration of Helsinki and Ethics Committee approval (EA2/162/17) was granted.

### 2.2 Clinical Assessment and Blood Sampling

In the paediatric cohort, blood was drawn during routine venipuncture in 3.2% trisodium citrate and K3 EDTA tubes. Study participants were assigned to pseudo‐anonymous number codes, and clinical data, including anthropometry, medical history, routine laboratory values and treatment regimen, were documented in a clinical database. Percentiles for height, weight and body mass index were obtained from national German references (Neuhauser et al. 2013). Blood pressure references were taken from the American National High Blood Pressure Education Program Working Group on High Blood Pressure in Children and Adolescents due to more specific scaling (National High Blood Pressure Education Program Working Group on High Blood Pressure in, C., and Adolescents 2004). eGFR was calculated using the Schwartz equation (Schwartz et al. 1987). Left ventricular hypertrophy was assessed by routine echocardiography.

In case of adult haemodialysis patients, blood was drawn in 3.2% trisodium citrate at the beginning and at the end of a dialysis session. Sample processing and clinical data collection were similar to the paediatric cohort.

### 2.3 Blood Sample Processing

Citrated blood samples were processed for EV analysis using a three‐step centrifugation protocol as previously described (McVey et al. 2018). Cells were removed by an initial centrifugation at 600 × *g* for 15 min, followed by two centrifugation steps at 2500 × *g* for 15 min each to remove residual cells and debris. Platelet‐free plasma was then snap frozen in liquid nitrogen and stored at −80°C.

EDTA plasma was obtained by centrifugation at 2500 × *g* for 10 min and stored at −80°C for targeted metabolomics.

### 2.4 Characterization of Patient EVs

EVs from citrated plasma were characterized by transmission electron microscopy (TEM), nanoparticle tracking analysis (NTA), flow cytometry, sphingolipidomics, small RNA sequencing and microRNA (miRNA) RT‐qPCR. Plasma was used without further purification for flow cytometry, size exclusion chromatography (SEC) was performed for NTA (qEVoriginal 70 nm columns, Izon Science, Christchurch, New Zealand), and membrane affinity‐based purification was used for TEM, sphingolipid and miRNA analyses (exoEasy Maxi Kit/exoRNeasy Midi Kit, Qiagen, Hilden, Germany).

Negative staining TEM was performed using purified EVs from 500 µL plasma concentrated to 100 µL using Vivaspin 2 columns with 100 kD molecular weight cut‐off (Sartorius, Göttingen, Germany) and fixed in 2% paraformaldehyde. For negative staining, 15 µL EVs were applied to parafilm and carbon‐coated EM grids were placed on top for 15 min. After washing, contrast was achieved by applying 1% uranyl acetate for 20 s. Images were captured on a Leo EM 906 (Carl Zeiss, Oberkochen, Germany, 27,800×).

Immunoblotting for CD9, CD41 and GAPDH was performed on EVs isolated from 100 µL plasma. Protein was extracted with non‐reducing sodium dodecyl‐sulphate (SDS) Laemmli buffer and 20 µg were run on 1.5 mm 10% bis(2‐hydroxyethyl)amino‐tris(hydroxymethyl)methane (Bis‐Tris) gels. Protein was transferred to a 0.2 µm polyvinylidene fluoride (PVDF) membrane and blocked with 5% non‐fat milk for 1 h. The membrane was incubated with primary antibodies overnight and secondary antibodies in 5% BSA for 2 h.

NTA was performed on a NanoSight LM20 (NanoSight, Amesbury, UK) equipped with a 632 nm laser using EVs from 50 µL plasma eluted in 1.5 mL PBS after SEC. Five measurements of 60 s each were performed per sample.

For EV flow cytometry, 4 µL of plasma was used for each of the four stainings, all containing FITC Annexin V and two antibodies (MiFlowCyt‐EV guideline report in Table S1, full antibody list in Table S2): (i) CD41 (BV510) + CD235a (PerCP/Cy5.5), (ii) CD14 (BV421) + CD31 (BV711), (iii) CD3 (BV421) + CD20 (BV605), (iv) CD66b (BV421) + CD68 (BV785). Annexin V staining was used as an internal control, but EVs were quantified independently of Annexin V. Samples were run on a BD Influx Cell Sorter (BD Biosciences, Franklin Lakes, NJ, USA) equipped with a 200 mW 488 nm laser, a 45 mW 405 nm laser and small particle optics. Counting beads were used for quantification.

LC–MS/MS sphingolipidomics was performed on EVs purified from 100 µL of plasma. Lipids were extracted with methanol/chloroform (2:1, v:v) as previously described (Gulbins et al. 2018). C17 ceramide and d_31_‐C16 sphingomyelin (both Avanti Polar Lipids, Alabaster, USA) were used as internal standards and chromatographic separations were achieved on a 1290 Infinity II HPLC equipped with a Poroshell 120 EC‐C8 column (3.0 × 150 mm, 2.7 µm; both Agilent Technologies, Waldbronn, Germany). MS/MS analyses were performed on a 6495C triple‐quadrupole mass spectrometer (Agilent Technologies) operating in the positive electrospray ionization mode and ceramides and sphingomyelins were quantified by multiple reaction monitoring. Sphingolipid concentrations were normalized to protein levels (as determined by the Bradford assay).

EV RNA was isolated from 500 µL of EVs and eluted in 12 µL RNase‐free water. 7 µL of RNA was used to generate cDNA libraries for small RNA sequencing (SMARTer smRNA‐Seq Kit for Illumina, Takara Bio USA, San Jose, CA, USA). Libraries were quality checked for base pair length (∼172 bp) using a fragment analyser (Advanced Analytical Technologies, Heidelberg, Germany). Sequencing was performed on a MiSeq sequencer using the MiSeq Reagent Kit v3 (150‐cycle, both Illumina, San Diego, CA, USA). Taqman Advanced miRNA Assays were used for RT‐qPCR validation of candidate miRNAs (Thermo Fisher Scientific). Detailed methods are provided in the supplemental material.

### 2.5 Functional EV Profiling

The biological functions of plasma EVs from the CKD cohort were analysed in vitro. Human aortic endothelial cells (HAoECs, PromoCell, Heidelberg, Germany) were incubated with patient EVs at a final dilution of 1:4 with plasma (healthy donors, HD, KTx, purified with exoEasy Maxi Kit, Qiagen) for 18 h. HAoECs were chosen to mimic endothelial exposure to CKD EVs in areas where CVD processes such as atherosclerosis occur. Bulk RNA sequencing of the cells (Qiazol (Qiagen) chloroform‐based RNA isolation) was performed on a NextSeq 2000 using the NextSeq 2000 P3 reagents (100 cycles, both Illumina) to identify transcriptomic changes in endothelial cells upon exposure to patient EVs.

Angiogenesis, specifically vascular tube formation, migration and proliferation of human umbilical vein endothelial cells (HUVECs, obtained shortly after isolation (Maroski et al. 2011)), and vascular smooth muscle cell (SMC, human aortic SMCs, PromoCell) proliferation were tested upon exposure to patient EVs at a final dilution of 1:9 compared to plasma (healthy donors and HD EVs purified using Exo‐spin mini‐columns, Cell Guidance Systems, Cambridge, UK). Angiogenic properties were tested in HUVECs, as the relevant assays are established in these cells, which have high angiogenic and proliferative properties. Tube formation and migration were assessed by light microscopy (EVOS M5000 microscope, Thermo Fisher Scientific, 10×) at 6 and 4 h, respectively. Proliferation was measured by the proportion of Ki67^+^ nuclei after 18 h of EV exposure, as assessed by immunofluorescence (EVOS M5000 microscope, Thermo Fisher Scientific, 20×–40×). Detailed methods are provided in the supplemental material.

### 2.6 Mechanisms of EV Release

Targeted tryptophan (TRP) metabolomics of plasma from the CKD cohort was performed as previously described (Holle et al. 2022) to identify associations of plasma TRP metabolites with EV populations. Carboxyfluorescein succinimidyl ester (CFSE)‐labelled HAoECs were exposed to candidate metabolites (indoxyl sulphate (IS), xanthurenic acid (XA, both Sigma‐Aldrich, St. Louis, MO, USA), formyl kynurenine (FKYN, Biozol, Eching, Germany)) in vitro for 2–4 h and concentrations of CFSE^+^ were measured by flow cytometry (BD Influx). The additive effects of indoxyl sulphate (IS) and high shear stress on EV release were tested in vitro by culturing CFSE‐labelled human saphenous vein endothelial cells (HSaVECs, PromoCell) under different flow conditions (Ibidi pump system with μ‐slides I 0.6 Luer, 1 dyn/cm^2^ for venous flow, 10 dyn/cm^2^ for arterial flow) with or without additional IS exposure (50 µM) for 18 h, followed by measurement of CFSE^+^ EVs by flow cytometry and EV miRNA cargo by RT‐qPCR. HSaVECs were chosen for this assay to mimic conditions on the venous side of arteriovenous fistulas (AVF) in HD patients. Similarly, the effect of 50 µM IS exposure (3 or 4 h respectively) was also tested on human platelets and monocyte‐derived macrophages. CD41^+^ or CD68^+^ EVs were measured by flow cytometry respectively and EV miRNA RT‐qPCR was performed.

### 2.7 Statistical Analyses

GraphPad Prism (GraphPad Software, San Diego, CA USA) was used for statistical analyses of NTA, flow cytometry, RT‐qPCR, in vitro results, comparisons of individual TRP metabolites and correlations of EC‐EVs with TRP metabolites. Kruskal–Wallis test and Dunn's post hoc test for non‐normally distributed data or one‐way ANOVA and Sidak's post hoc test for normally distributed data were used for multiple comparisons of cross‐sectional data, Mann–Whitney *U* test for single comparisons and Wilcoxon or paired Student's *t*‐test for longitudinal data, as appropriate. *p* < 0.05 was considered statistically significant. Normal distribution was tested with the Kolmogorov–Smirnov test.

Differentially enriched EV sphingolipids and plasma TRP metabolites from lipidomics and metabolomics were identified using Wilcoxon rank‐sum tests with FDR correction for multiple testing. Differentially enriched miRNAs were identified from EV small RNA sequencing data using DESeq2 (Love et al. 2014) and confirmed using LongDat (Chen et al. 2023). DESeq2 was also used to identify differentially enriched genes in HAoEC transcriptomics after treatment with patient EVs. FDR corrected *p* values < 0.1 were considered significant.

## 3 Results

### 3.1 Plasma Extracellular Vesicles Are Increased in Patients on Peritoneal Dialysis

**FIGURE 1:**
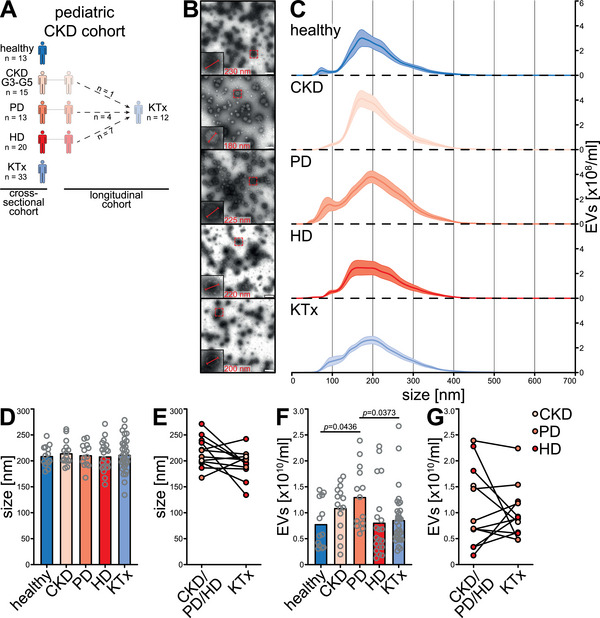
Plasma extracellular vesicle concentrations increase in peritoneal dialysis patients. (A) 94 children and adolescents were enrolled in the paediatric CKD study, which consisted of five groups in the cross‐sectional study: (i) healthy donors, (ii) CKD G3‐G5 patients not requiring kidney replacement therapy, (iii) patients on peritoneal dialysis (PD) or (iv) haemodialysis (HD) and (v) patients who had already received a kidney transplant (KTx) and had stable graft function. During the study period 12 patients underwent KTx and longitudinal follow‐up after KTx was performed. (B) EVs of similar shape and size could be visualized in the plasma of all CKD patient groups by negative staining transmission electron microscopy, scale bar 400 nm. Nanoparticle tracking analysis (NTA) revealed that plasma EV size was not affected by CKD, as shown by (C) similar size distribution (data given as mean of all patients ± SEM), (D) similar mean EV size and (E) no changes in EV size after KTx. (F) Total plasma EV concentrations were increased in PD patients as compared to healthy donors and KTx recipients. (G) Longitudinal EV concentration did not change after KTx. HD haemodialysis patients. *P* values according to Kruskal–Wallis test and Dunn's post hoc test.

To investigate the impact of EVs in CKD, we performed an in‐depth characterization of plasma EVs in a single‐centre cross‐sectional study enrolling 94 children, including 13 healthy donors, 15 CKD G3‐G5 patients, 13 PD patients, 20 HD patients and 33 KTx patients (Figures 1A and S1). The mean age was 10.9 ± 0.5 years and 49% of the participants were female. Patients after KTx showed stable graft function (eGFR 104.5 ± 5.6 mL/min/1.73 m2) at 45.3 ± 7.8 months after KTx (Table 1). In 12 CKD patients longitudinal follow‐up after KTx was performed allowing intra‐individual comparisons of EV parameters with impaired and restored kidney function (Figure 1A and S1). Here, the mean time after KTx was 5.0 ± 1.1 months and eGFR normalized to 94.5 ± 5.2 mL/min/1.73 m2 (Table 2). All CKD groups had significant cardiovascular morbidity, as evidenced by arterial hypertension (79%–95% of patients in the respective groups) and left ventricular hypertrophy (13%–35%, Table 3). Resistant hypertension, defined as systolic or diastolic blood pressure above the 95th percentile despite treatment with three or more antihypertensive drugs at adequate dose (Carey et al. 2018), was more frequent in CKD, PD and HD than in patients after KTx (27%–38% vs. 12%, Table 3).

**TABLE 1:** Baseline clinical characteristics of the cross‐sectional cohort. Data are presented as mean ± SEM or absolute values and percentages where appropriate.

	Healthy	CKD G3‐G5	PD	HD	KTx	All
Individuals (*N*)	13	15	13	20	33	94
Age (years)	12.5 ± 1.4	10.1 ± 0.9	5.9 ± 1.4	13.9 ± 0.7	10.8 ± 0.6	10.9 ± 0.5
Female	9 (69%)	6 (40%)	5 (38%)	11 (55%)	15 (45%)	46 (49%)
Kidney diagnosis						
CAKUT	0	6 (40%)	3 (23%)	6 (30%)	14 (42%)	29 (31%)
Tubulointerstitial	0	1 (7%)	0	0	1 (3%)	2 (2%)
Post‐AKI	0	1 (7%)	1 (8%)	1 (5%)	0	3 (3%)
Glomerulopathy	0	1 (7%)	3 (23%)	4 (20%)	6 (18%)	14 (15%)
Cystic	0	4 (27%)	2 (15%)	5 (25%)	7 (21%)	18 (19%)
Other	0	2 (13%)	4 (31%)	4 (20%)	5 (15%)	15 (16%)
Healthy	13 (100%)	0	0	0	0	13 (14%)
Time since KTx (months)	N/A	N/A	N/A	N/A	45.3 ± 7.8	N/A
Creatinine (mg/dL)	0.6 ± 0.1	3.3 ± 0.5	8.4 ± 1.1	8.7 ± 0.6	0.9 ± 0.1	4.0 ± 0.4
eGFR (mL/min/1.73 m2)	148.7 ± 7.9	31.8 ± 4.6	6.9 ± 0.3	10.7 ± 0.7	104.5 ± 5.6	64.7 ± 5.9
BUN (mg/dL)	12.1 ± 0.2	51.0 ± 4.7	43.9 ± 6.2	57.0 ± 3.7	21.5 ± 1.8	35.8 ± 2.3
Uric acid (mg/dL)	4.3 ± 0.4	6.9 ± 0.3	6.2 ± 0.6	6.5 ± 0.5	5.4 ± 0.5	5.8 ± 0.2
Phosphate (mmol/L)	1.4 ± 0.1	1.6 ± 0.1	1.7 ± 0.2	1.6 ± 0.1	1.5 ± 0.0	1.6 ± 0.0
Albumin (g/L)	45.7 ± 0.7	41.6 ± 1.5	33.1 ± 1.4	38.6 ± 1.2	41.3 ± 1.1	40.1 ± 0.7
PTH (pmol/L)	N/A	27.2 ± 8.0	54.6 ± 16.2	35.7 ± 6.7	10.4 ± 2.4	N/A
Triglycerides (mg/dL)	101.1 ± 12.0	164.2 ± 19.9	269.6 ± 55.2	132.0 ± 12.8	164.7 ± 25.7	163.8 ± 14.4

Abbreviations: AKI, acute kidney injury; BUN, blood urea nitrogen; CAKUT, congenital anomalies of the kidney and the urinary tract; eGFR, estimated glomerular filtration rate based on serum creatinine and the Schwartz equation; PTH, parathyroid hormone.

**TABLE 2:** Baseline clinical characteristics of patients with longitudinal follow‐up after kidney transplantation. Data are presented as mean ± SEM or absolute values and percentages where appropriate.

	CKD G5	KTx
Individuals (*N*)	12	
Age (years)	10.7 ± 1.1	11.8 ± 1.1
Female	6 (50%)	
Kidney diagnosis		
CAKUT	3 (25%)	
Tubulointerstitial	0	
Post‐AKI	0	
Glomerulopathy	2 (16.7%)	
Cystic	3 (25%)	
Other	4 (33.3%)	
PD	4 (33.3%)	0
HD	7 (58.3%)	0
Time since KTx (weeks)	N/A	5.0 ± 1.1
Creatinine (mg/dL)	8.1 ± 0.7	0.8 ± 0.1
eGFR (mL/min/1.73 m2)	9.9 ± 1.0	94.5 ± 5.2
BUN (mg/dL)	56.7 ± 4.3	21.4 ± 2.0
Uric acid (mg/dL)	6.7 ± 0.9	5.4 ± 0.6
Phosphate (mmol/L)	1.7 ± 0.2	1.5 ± 0.1
Albumin (g/L)	37.2 ± 1.5	40.3 ± 1.9
PTH (pmol/L)	52.5 ± 13.0	13.1 ± 5.1
Triglycerides (mg/dL)	258.4 ± 73.4	209.5 ± 57.5

Abbreviations: AKI, acute kidney injury; BUN, blood urea nitrogen; CAKUT, congenital anomalies of the kidney and the urinary tract; eGFR, estimated glomerular filtration rate based on serum creatinine and the Schwartz equation; PTH, parathyroid hormone.

**TABLE 3:** Cardiovascular morbidity in the cross‐sectional cohort. Data are presented as mean ± SEM or absolute values and percentages where appropriate. Resistant hypertension was defined as systolic or diastolic blood pressure above the 95th percentile despite treatment with three or more antihypertensive drugs at adequate doses.

	Healthy	CKD G3‐G5	PD	HD	KTx	All
Hypertension	0	12 (80%)	12 (93%)	19 (95%)	26 (79%)	69 (73%)
N° of anti‐hypertensives	0	1.5 ± 0.4	1.4 ± 0.4	2.2 ± 0.4	1.4 ± 0.2	1.4 ± 0.2
Resistant hypertension	0	4 (27%)	5 (38%)	6 (30%)	4 (12%)	19 (20%)
LV hypertrophy	N/A	2 (13%)	3 (23%)	7 (35%)	6 (18%)	N/A

Abbreviation: LV, left ventricle.

Plasma EV isolation was validated and TEM showed the presence of EVs of comparable shape and size in all patient groups (Figure 1B). Moreover, immunoblots showed the presence of CD9, CD41 and GAPDH on all nine representative EV isolates from the respective patient groups (Figure S2). Enabling label‐free absolute quantification of EVs at high size resolution, NTA showed similar mean EV size in all patients (Figure 1C–E), with higher total concentrations in PD patients compared to healthy donors and KTx patients (Figure 1F). Total EV concentrations did not change longitudinally after KTx (Figure 1G).

### 3.2 Endothelial EVs Are Increased in Haemodialysis Patients, Whereas T Cell‐ and Macrophage‐Derived EVs Are Increased in CKD Patients Without Dialysis

**FIGURE 2:**
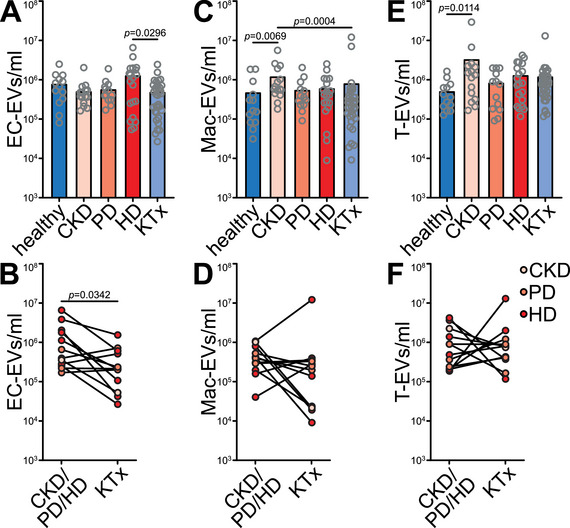
CD31^+^ endothelial EVs are increased in haemodialysis patients, whereas CD3^+^ T cell and CD68^+^ macrophage EVs are increased in CKD patients without dialysis. Specialized multicolour EV flow cytometry was performed on plasma from all patients in the paediatric CKD cohort. Cross‐sectional (A) and longitudinal (B) comparison of EVs of different cellular origin: CD31^+^ endothelial (EC‐) EVs were higher in haemodialysis (HD) patients as compared to kidney transplant (KTx) recipients in cross‐sectional comparison and decreased in longitudinal follow‐up of CKD patients after receiving KTx. CD68^+^ macrophage‐derived (Mac‐) EVs and CD3^+^ T cell‐derived (T‐) EVs were both increased in CKD without dialysis as compared to healthy donors and, in the case of Mac‐EVs, also compared to KTx recipients. Longitudinal analyses showed no significant changes in Mac‐EVs and T‐EVs. PD peritoneal dialysis patients. *P* values according to (A) Kruskal–Wallis test and Dunn's post hoc test, (B) Wilcoxon test.

To analyse changes in circulating hematopoietic and EC‐EVs, multiparametric flow cytometry was applied to platelet‐free plasma, accounting for eight cell lineages (Figure S3). CD41^+^ platelet‐derived (Plt) EVs were the most abundant EV fraction, but remained unchanged in CKD cross‐sectionally and in longitudinal follow‐ups, as did EVs of B cell, monocyte, neutrophil and erythrocyte origin (Figure S4). The concentration of EC‐EVs was increased 3‐fold in HD patients as compared to KTx recipients cross‐sectionally (Figure 2A), and a longitudinal decrease in EC‐EVs was observed in patients after KTx and was most pronounced in HD patients who had high EC‐EV concentrations before KTx (Figure 2B). Concentrations of CD68^+^ macrophage‐derived (Mac‐) EVs and CD3^+^ T cell (T‐) EVs were increased 3‐ and 6‐fold respectively in CKD patients without dialysis compared to healthy donors, and Mac‐EVs were normalized in KTx patients to levels comparable to healthy donors (Mac‐EVs, Figure 2A). Mac‐ and T‐EVs did not change longitudinally (Figure 2B).

In conclusion, the composition of plasma EVs is influenced by the stage of CKD. KTx patients showed a similar composition to healthy controls. Furthermore, we observed CKD effects on Mac‐ and T‐EVs. Increased EC‐EV levels as observed in patients on HD, may be of interest given the established association between EC‐EVs and CVD (Behrens et al. 2020) and increased cardiovascular risk in dialysis patients. In addition to their cellular origin, the cargo of EVs is of central interest for their biological function.

### 3.3 CKD Alters the MicroRNA Profile of Extracellular Vesicles

**FIGURE 3:**
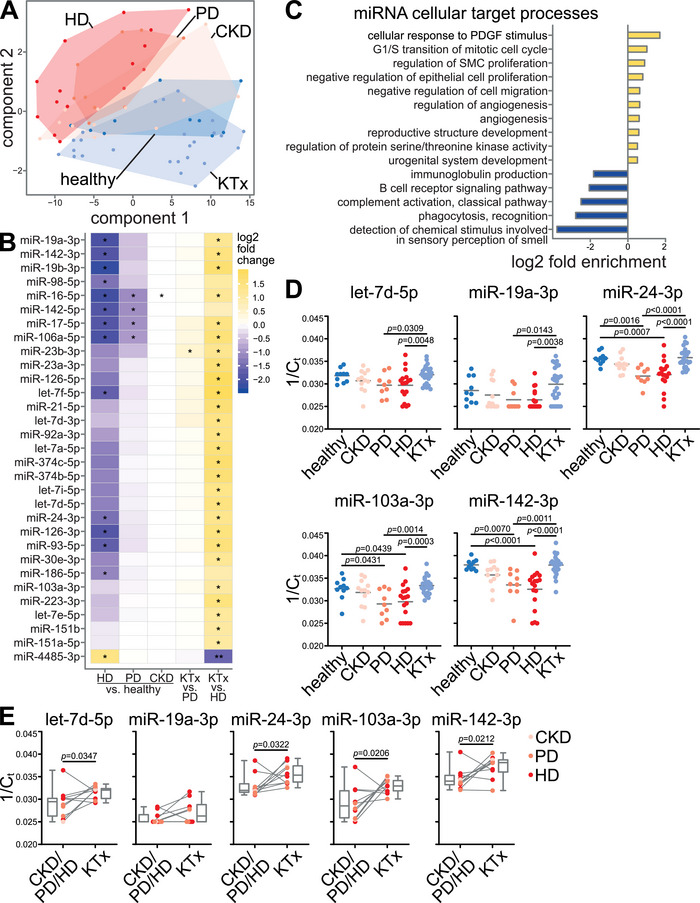
EV microRNA cargo is altered in CKD patients on dialysis and restored after kidney transplantation. Small RNA sequencing was performed on EVs from 75 patients of the paediatric CKD cohort. (A) Partial least‐squares discriminant analysis (PLS‐DA) showed a clear differentiation of EV microRNAs (miRNAs) between patients with normal kidney function (healthy donors, kidney transplant recipients (KTx)), and dialysis patients (haemodialysis (HD) and peritoneal dialysis (PD) patients) with CKD without dialysis in between, corresponding to the clinical state of intermediate reduced kidney function. (B) 31 miRNAs were differentially regulated in CKD, PD and/or HD patients as compared to healthy donors and/or KTx patients as shown in the heatmap of group comparisons according to DESeq2 for miRNAs that were also confirmed by LongDat. (C) Cellular target processes of altered miRNAs in CKD patients were predicted using TargetScanHuman miRNA target gene identification and PANTHER gene set enrichment analyses. Most specific gene ontology (GO) terms of hierarchical trees with fold enrichment ≥1.5 or ≤0.5 and FDR‐corrected *p* < 0.05 according to Fisher's exact test are shown. (D) RT‐qPCR confirmed that five selected miRNAs were reduced in the EVs of dialysis patients and (E) KTx partially reversed these changes in longitudinal comparison according to RT‐qPCR. (B) *p < 0.1, **p < 0.01 by DESeq2 with FDR‐correction. (D) *P* values according to one‐way ANOVA and Sidak's post hoc test for normally distributed data or Kruskal–Wallis test and Dunn's post hoc test for non‐normally distributed data as appropriate. (E) *P* values according to paired Student's *t*‐test for normally distributed data or Wilcoxon test for non‐normally distributed data.

To probe for a potential functional relevance of CKD EVs, we analysed the EV sphingolipid content as an EV cargo with potential functional relevance in CVD (Elsherbini and Bieberich 2018). Using lipidomics, no consistent changes were observed in CKD and HD patients compared to healthy controls, with the exception of an upregulation of C16 sphingomyelin (SM16) in CKD, but not HD (Figure S5). We therefore focused on microRNAs (miRNAs), which have been shown to be abundant in EVs and involved in post‐transcriptional gene regulation in target cells (Behrens et al. 2020; Raposo and Stoorvogel 2013). PLS‐DA of miRNA reads from EV small RNA sequencing revealed a clear distinction between individuals with normal kidney function (healthy donors, KTx patients) and those on dialysis. CKD patients without dialysis formed an intermediate cluster corresponding to their milder clinical phenotype (Figure 3A). Using DESeq2 (Love et al. 2014), we identified 32 altered miRNAs comparing CKD/PD/HD versus healthy/KTx (Figure S6A), of which 31 were validated using LongDat (Chen et al. 2023) (Figure S6B,C). Among these, 30 miRNAs showed reduced abundance in CKD patients, most pronounced in HD patients, compared to healthy donors, and increased in KTx recipients compared to dialysis patients, except for miR‐4485‐3p which showed higher levels in HD (Figure 3B).

### 3.4 Predicted Targets of MicroRNAs Regulated in CKD EVs Include Vascular Processes Dysregulated in Cardiovascular Disease

Next, we identified cellular target processes of the altered miRNAs in CKD EVs (see Figure S7A for a detailed description of the target identification protocol). Target genes of all 31 miRNAs were predicted using TargetScanHuman (Agarwal et al. 2015), yielding 4113 genes after filtering. Gene set enrichment of these genes identified 172 Gene Ontology (GO) terms, which were filtered for fold change ≥1.5 or ≤0.5 and specificity, resulting in 15 predicted GO terms, of which ten were enriched and five were underrepresented (Figure 3C). Enriched GO terms included several cellular processes dysregulated in CVD pathophysiology, that is, ‘cellular response to platelet‐derived growth factor stimulus’, ‘regulation of SMC proliferation’, ‘regulation of angiogenesis’ and ‘angiogenesis’. For RT‐qPCR validation, the five dysregulated miRNAs with the highest probability of interacting with genes of enriched GO terms were selected (Figure S7B). Among them, miR‐24‐3p, miR‐103a‐3p and miR‐142‐3p were cross‐sectionally reduced in PD and HD patients compared to healthy donors with restored levels after KTx, whereas let‐7d‐5p and miR‐19a‐3p were higher in KTx compared to PD and HD but not lower in dialysis compared to healthy donors (Figure 3D). Longitudinal analyses confirmed a significant increase for let‐7d‐5p, miR‐24‐3p, miR‐103a‐3p and miR142‐3p after KTx (Figure 3E). miR‐4485‐3p as the only miRNA upregulated in HD according to sequencing was not detectable by RT‐qPCR. These findings suggest that circulating EVs in CKD patients contain lower levels of specific miRNAs compared to healthy individuals with potential functional relevance in CVD processes and reversal of EV miRNA dysregulation after KTx.

### 3.5 CKD EVs Impair Endothelial Angiogenic Properties in Vitro

**FIGURE 4:**
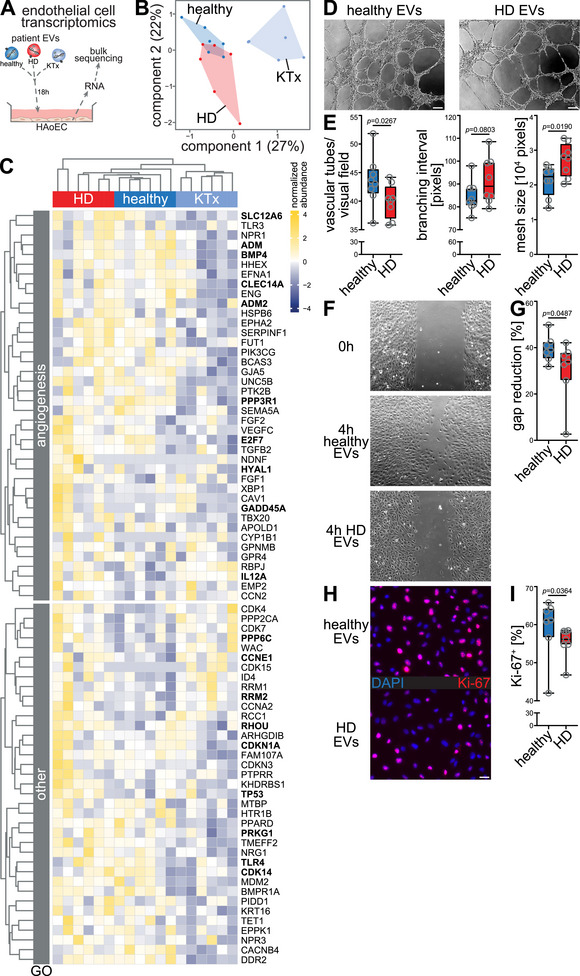
CKD EVs cause impaired angiogenesis at transcriptomic and functional levels. The effect of plasma EVs from healthy donors and CKD patients on endothelial function was assessed using bulk RNA sequencing of human aortic endothelial cells (HAoECs) and functional assays on human umbilical vein endothelial cells (HUVECs). (A) HAoECs were incubated with isolated plasma EVs from healthy donors, hemodialysis (HD) patients and kidney transplant (KTx) recipients for 18 h and RNA was isolated from HAoECs for bulk RNA sequencing. (B) Principal component analysis (PCA) of genes belonging to gene ontology (GO) terms ‘cellular response to platelet‐derived growth factor stimulus’ (GO:0036120), ‘G1/S transition of mitotic cell cycle’ (GO:0000082), ‘regulation of smooth muscle cell proliferation’ (GO:0048660), ‘regulation of angiogenesis’ (GO:0045765), ‘negative regulation of cell migration’ (GO:0030336) and ‘angiogenesis’ (GO:0001525), which were identified by microRNA target prediction, showed a clear separation of healthy donors, HD and KTx patients. (C) Heatmap of differentially enriched genes (DEGs) of these GO terms showed an independent clustering of patient groups with the most pronounced changes between HD and KTx patients and the majority of DEGs involved in angiogenesis. Differential abundance was tested using DESeq2 with FDR‐correction. Based on the prediction of miRNA targets and transcriptomic changes in HAoECs upon CKD EV treatment functional angiogenesis properties were tested in vitro. Matches of DEGs with miRNA target genes are highlighted in bold. HUVECs were seeded on Matrigel and incubated with healthy or HD EVs for 6 h and vascular tube formation was analysed. (D) Representative microscopic images of HUVECs after 6 h of incubation. (E) Quantification of angiogenic properties: manual quantification of vascular tube‐like structures showed decreased angiogenesis of HD EV‐treated cells, which was supported by automated quantification using *ImageJ Angiogenesis Analyzer* software indicating decreased vessel density upon HD EV treatment as compared to healthy EVs, as evidenced by an increased branching interval, although without reaching statistical significance, and significantly increased mesh size. Vascular tube formation results were confirmed by assessing endothelial (HUVEC) (F,G) migration and (H,I) proliferation (immunofluorescence for Ki‐67). (F) HUVECs were grown with cell culture inserts to obtain a standardized gap area. Representative microscopic images of HUVECs before (0 h) and after incubation with healthy/HD EVs (4 h), (G) reduced relative reduction of gap area upon HD EV exposure as compared to healthy EVs. (H) Representative microscopic images of proliferating HUVECs after incubation with healthy/HD EVs for 18 h, (I) reduced endothelial proliferation upon exposure to HD EVs compared to healthy EVs as measured by the proportion of Ki67^+^ nuclei. Scale bars (D,F) 100 µm (10×), (H) 25 µm (40×). *P* values according to Mann–Whitney *U* test.

To assess the functional relevance of CKD EVs on the vasculature, we exposed HAoECs to EVs from healthy donors, HD and KTx patients for 18 h and performed bulk RNA sequencing of these cells after EV treatment (Figure 4A). Among the six GO terms related to vascular homeostasis predicted by miRNA target identification, targeted gene analysis revealed patient group‐specific clustering, with the strongest dysregulation of genes belonging to the GO term ‘angiogenesis’ (Figure 4B,C). Ten out of 40 differentially regulated genes related to angiogenesis were targets of differentially regulated miRNAs in CKD EVs, suggesting a functional relevance of dysregulated EV miRNAs for altered expression of angiogenesis genes (Figures 4C and S8). Therefore, we next probed for the functional effect of CKD EVs on angiogenesis in vitro. HUVECs treated with EVs from HD patients showed reduced vascular tube formation compared to healthy EV‐treated cells resulting in decreased vessel density as indicated by increased mesh size and a trend towards higher branching intervals (Figure 4D,E). In addition, HD EV‐treated HUVECs showed impaired migration (Figure 4F,G) and proliferation (Figure 4H,I) compared to healthy EV‐treated cells. Aortic SMCs did not show comparable changes in proliferation (Figure S9). As such, EV miRNAs may be associated with functional EV effects on the vasculature, potentially driving phenotypic changes such as capillary rarefaction that are well described in CKD (Steegh et al. 2023).

### 3.6 Accumulation of Uremic Tryptophan Metabolites in CKD in Combination With Mechanical Stress Triggers Endothelial EV Release

**FIGURE 5:**
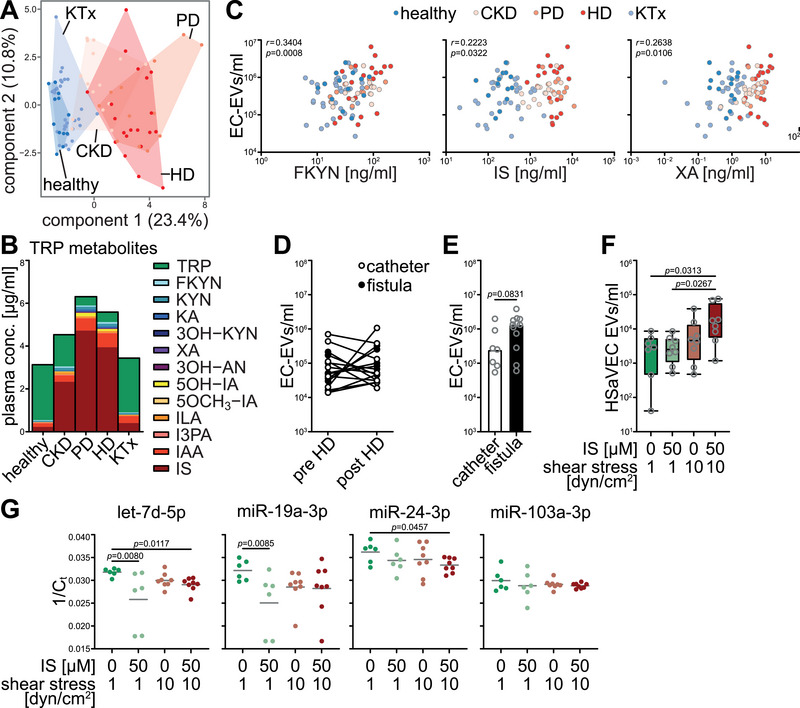
Dysregulated tryptophan metabolism induces endothelial EV release during vascular mechanical stress. To assess the role of tryptophan (TRP)‐derived uremic toxins in EV release in CKD targeted plasma metabolomics from the paediatric CKD cohort was performed. (A) Principal component analysis (PCA) clearly differentiated patients with normal kidney function (healthy donors and kidney transplant (KTx) recipients) from dialysis patients (HD haemodialysis, PD peritoneal dialysis) with CKD without dialysis in between. (B) Cumulated mean plasma concentrations show a major shift of TRP metabolism towards decreased TRP levels and increased indole metabolites in CKD, most prominently indoxyl sulphate (IS) with highest concentrations in dialysis patients. (C) Formyl kynurenine (FKYN), IS and xanthurenic acid (XA) correlated positively with endothelial EV (EC‐EV) plasma concentrations. To address potential trigger factors for increased endothelial EV release in HD patients two potential mechanisms were analysed. (D) Acute effects of HD on EV release were analysed in an additional cohort of CKD patients comparing EV concentrations before and after one HD session with no significant changes in endothelial (EC‐) EV concentrations. (E) The influence of dialysis access was addressed comparing HD patients with central venous catheters and patients with arteriovenous fistula (AVF) in the original paediatric CKD cohort, which showed a higher mean EC‐EV concentration in patients with AVF, but without reaching statistical significance. To evaluate the role of abnormal arterial flow conditions with high shear stress on the venous side of the AVF in HD human saphenous vein endothelial cells (HSaVECs) in vitro under different flow and uraemia conditions in μ‐slides using a pumping system to achieve unidirectional laminar flow. (F) Flow cytometry showed increased EV release from HSaVECs as measured by CFSE staining under dual stimulation with arterial high shear stress (10 dyn/cm^2^) and uremic 50 µM indoxyl sulphate (IS) as compared to venous low shear stress (1 dyn/cm^2^) ± 50 µM IS. (G) EV miRNA RT‐qPCR from these experiments showed that the combination of high shear stress with uremic stimulus was sufficient to partially recapitulate the EV miRNA reduction observed in CKD patients, as indicated by significantly lower abundance of let‐7d‐5p and miR‐24‐3p in 10 dyn/cm^2^ + 50 µM IS as compared to 1 dyn/cm^2^ + vehicle, while 50 µM IS alone was also sufficient to lower let‐7d‐5p and miR‐19a‐3p. IAA indoleacetic acid, ILA indole lactate, I3PA indole‐3‐propionic acid, KA kynurenic acid, KYN kynurenine, TRP tryptophan, 3OH‐AN 3OH‐anthranilate, 5OCH_3_‐IA 5OCH_3_‐indoleacetate, 5OH‐IA 5OH‐indoleacetate. (C) *r* and *p* according to Pearson's correlations. (E) *P* value according to Mann–Whitney *U* test. (F,G) *P* values according to Kruskal–Wallis test and Dunn's post hoc test.

Finally, we aimed to elucidate potential mechanisms of EV release in CKD. In a first set of experiments, we focused on EC‐EVs, as these are significantly increased in HD patients and have previously been implicated in CVD (Buffolo et al. 2022). We suspected microbiome‐derived uremic toxins as potential mediators. To this end, targeted metabolomic analyses of TRP metabolites were performed as prototypic uremic toxins. As previously shown (Holle et al. 2022) TRP metabolites discriminated between individuals with normal kidney function (healthy donors, KTx patients) and CKD patients in a stage‐dependent manner (Figure 5A). Total TRP metabolite concentrations were elevated in CKD, with the two most prominent metabolites, TRP and IS, showing an inverse relationship with decreased TRP and increased IS (Figures 5B, S10, S11). Three metabolites—XA, FKYN and IS—correlated with EC‐EV counts in the cross‐sectional cohort (Figure 5C, Table S4). However, when we treated HAoECs with increasing concentrations of exogenous XA, FKYN or IS in vitro, no detectable effect on EV release was observed (Figure S12).

Considering that EC‐EVs were increased in patients on HD, we investigated whether HD treatment could also affect the acute release of EVs. In adult HD patients (Table S5), EC‐EVs did not change as a function of dialysis, suggesting that HD itself does not trigger EV release (Figure 5D). However, a subgroup analysis of EC‐EVs concentrations by vascular access in paediatric HD revealed a 2.5‐fold higher mean EC‐EV concentration in individuals with an AVF compared to a central venous catheter (Figure 5E).

We therefore hypothesized that increased vascular shear stress acting on venous endothelial cells—either alone or in combination with TRP metabolites—may trigger EC‐EV release. We cultured HSaVECs under venous or arterial shear stress and exposed them to vehicle control or IS under constant flow for 18 h. We specifically focused on IS as previous studies have suggested its role in vascular remodelling (Arinze et al. 2022; Nakano et al. 2019) and as IS levels correlate with CVD in paediatric CKD (Holle et al. 2019). High shear stress alone did not induce additional EV formation, yet, in HSaVECs treated with high shear stress in combination with IS EV release increased compared to the low shear stress control (Figure 5F). We next investigated whether EC‐EVs generated under high shear stress and IS mimic the cargo of CKD EVs from our in vivo study. Of the five miRNAs identified in patients, four were detectable in HSaVEC‐EVs by RT‐qPCR. Let‐7d‐5p and miR‐24‐3p were reduced by high shear stress and IS, whereas miR‐19a‐3p did not reach statistical significance (Figure 5G). Of note, the addition of IS alone already affected the miRNA cargo, as shown by the significant reduction of 2 out of 4 miRNAs detected.

Since our data indicate that IS alone already affected the miRNA cargo of EC‐EVs similar to the phenotype in vivo in total EVs, we investigated whether EVs from other cell types were similarly affected by IS. Since Plt‐EVs are the major fraction of circulating EVs (Figure S4) and Mac‐EVs were already altered in their abundance in earlier stages of CKD, we focused on these cell types and analysed EV release and cargo after incubation with IS. Similar to ECs, IS alone did not result in increased EV release in either platelets or macrophages (Figure S13A,B). Furthermore, no significant effect of IS on Plt‐ or Mac‐EV miRNA cargo was observed (Figure S13C,D).

In conclusion, our data implicate shear stress in combination with IS as a driver of EC‐EV release in advanced CKD. Moreover, the analysis of miRNA cargo suggests a contribution to the in vivo observation in total circulating EVs. However, the proposed mechanism is exclusively found in endothelial cells as compared to macrophages or platelets. Future studies are needed to uncover additional mechanisms of EV release and cargo alteration in CKD.

## 4 Discussion

Given the high prevalence of CKD (Jha et al. 2013) and the critical role of CVD in the survival of CKD patients (Matsushita et al. 2022), prevention of CVD in CKD is an unmet need of global importance. Here, we show that endothelial EV release is increased in children with advanced CKD. This is in line with previous studies in adult CKD patients reporting increased circulating EC‐EVs (Cavallari et al. 2019; Faure et al. 2006; Burton et al. 2013; Trappenburg et al. 2012; Ariza et al. 2013; Merino et al. 2010; Carmona et al. 2017; Soriano et al. 2014; Amabile et al. 2005; Esquivias‐Motta et al. 2017). However, in adults with CKD, CVD risk factors such as diabetes and hypertension often precede CKD (Matsushita et al. 2022). As these risk factors by themselves increase endothelial EV release (Preston et al. 2003; Pardo et al. 2018), a direct effect of CKD has so far been obscured. By analysing children with CKD, who develop CVD almost exclusively as a consequence of CKD (Mitsnefes 2012), we now demonstrate a direct and specific effect of CKD on EC‐EV release. Notably, we found increased EC‐EV levels only in HD patients, whereas some adult studies have also reported similar increases in CKD without dialysis and PD patients (Cavallari et al. 2019; Faure et al. 2006; Trappenburg et al. 2012; Merino et al. 2010). One study previously found a stage‐dependent increase in EC‐EVs in children with CKD without dialysis and those on dialysis (Dursun et al. 2009). Although our study did not confirm these findings at an earlier stage of the disease, we also found increased EC‐EV levels in HD patients in our cohort who had been on dialysis for longer than PD patients, highlighting that EC‐EVs may be mediators that emerge only after several years of disease. Alternatively, or rather, in parallel, endothelial EV release may also be affected by dialysis mode as—similar to our results—Dursun and colleagues found higher EC‐EV levels in HD than in PD patients despite shorter duration of kidney disease and time on dialysis in HD patients (Dursun et al. 2009) suggesting that HD‐related factors may specifically promote endothelial EV release.

Transcriptomic analysis of EV cargo revealed a reduction of several miRNAs in CKD EVs, especially in HD patients, but also patients on PD. We identified several cellular processes of functional relevance in CVD as potential targets of these dysregulated miRNAs. These data are consistent with a recent study reporting reduced abundance of several EV miRNAs—including miR‐16‐5p and miR‐17‐5p, which were also detected in our cohort—in rodents and adults with CKD (Koide et al. 2023). Moreover, two EV miRNAs have been identified as reduced in patients with CKD and coronary artery disease of which miR‐126‐3p overlaps with our findings (Zietzer et al. 2022). Taken together, these data are consistent with a phenotype of reduced abundance of specific EV miRNAs in CKD. In particular, our data from a paediatric cohort suggest that a reduction in distinct miRNAs may reflect an EV phenotype specific to CKD independent of comorbidities. Indeed, to our knowledge, a broad reduction of several EV miRNAs has not been reported in patients with other CVD risk factors such as hypertension or diabetes (Martinez‐Arroyo et al. 2021; Chao et al. 2023).

Bioinformatic analyses predicted angiogenesis and vascular SMC proliferation as potential targets of dysregulated EV miRNAs. This was experimentally confirmed as EVs from HD patients altered the transcriptome of endothelial cells in vitro compared to EVs from healthy donors/KTx patients, with the majority of differentially enriched genes related to angiogenesis. Among these genes, 25% were predicted targets of dysregulated miRNAs in CKD EVs, suggesting that an altered EV miRNA cargo may be a source of altered gene expression in the endothelium and subsequent impairment of angiogenesis. This interpretation is supported by our functional results showing that CKD EVs inhibit angiogenesis in vitro. Impaired angiogenesis is recognized as an early pathophysiological feature of CKD (Ravid et al. 2021; Jacobi et al. 2006; Prommer et al. 2018) and is considered to contribute to the microvascular rarefaction observed in CKD (Querfeld et al. 2020), ultimately leading to tissue hypoxia (Prommer et al. 2018; Goligorsky 2020), which in turn drives CVD processes such as vascular calcification (Negri 2023). As such, the proposed release of EC‐EVs, which inhibits angiogenesis via a loss of specific miRNAs may represent a relevant pathophysiological sequence in the development of CVD in CKD.

This view is consistent with data from Cavallari and coworkers who reported that CKD EVs may impair angiogenesis (Cavallari et al. 2019). Yet again, our data extend this finding to paediatric CKD thus highlighting its specificity for CKD. However, in contrast to Cavallari's work, which proposed an increase in miR‐223 in CKD EVs as a driving factor of impaired angiogenesis (Cavallari et al. 2019), we rather attribute this effect to a decrease of distinct EV miRNAs. Koide and colleagues further reported an important role of reduced EV miRNAs in vascular calcification in adult CKD (Koide et al. 2023), which was, however, not among the targets of the dysregulated miRNAs in our cohort and was, therefore, not tested, while SMC proliferation showed no difference after treatment with CKD EVs compared to healthy donor EVs. Taken together, previous data (Koide et al. 2023) and our study highlight that EVs potentially contribute to different processes of CVD development in CKD and suggest a reduced abundance of specific EV miRNAs as a common underlying pathophysiological mechanism.

To better understand the mechanisms of altered EV release in CKD, we investigated the role of uremic TRP metabolites. We found associations between three accumulated metabolites in CKD—FKYN, IS and XA—with plasma EC‐EV levels. However, functional testing, revealed no increase in EV release from primary aortic endothelial cells following exposure to these metabolites in vitro, in contrast to previous data reporting higher EC‐EV release from HUVECs following exposure to IS (Alique et al. 2020). Yet, in this previous work, only CD31^+^ Annexin V^+^ EVs were quantified, possibly reflecting the formation of apoptotic bodies due to high IS levels combined with long incubation times (Alique et al. 2020). In contrast, our study used shorter incubation times and quantified total CFSE^+^ EVs from HAoECs pretreated with CFSE.

Focusing specifically on the elevated EC‐EV levels in HD patients, we found that HD *per se* does not act as an acute trigger for endothelial EV release. While our data did not show consistent changes in EC‐EVs during HD, previous studies have shown mixed results. One study highlighted a slight decrease in several EV populations during HD, including EC‐EVs (Ruzicka et al. 2019), whereas others found an increase in EC‐EVs after HD (Liu et al. 2024; Ramirez et al. 2007) or, similar to our results, no changes of EC‐EVs during HD (Faure et al. 2006). Rather, we found a trend towards higher EC‐EV levels in patients with AVF as dialysis access compared to those with central venous catheters. This raised the question whether the disturbed flow conditions on the venous side of the fistula with unphysiologically high flow rates and shear stresses, might drive local endothelial EV release. While high shear stress alone was not sufficient to trigger EV release from venous endothelial cells in vitro, the combination of high shear stress with the uremic toxin IS increased EV formation, and in parallel recapitulated parts of the distinct changes in EV miRNAs observed in HD patients. This is consistent with previous studies reporting increased EV release upon high shear stress from other cell types (Sangha et al. 2023; Bratengeier et al. 2024). The specific molecular mechanisms of increased endothelial EV release remain to be elucidated, but may include activation of the aryl hydrocarbon receptor (AhR) by IS, which has been demonstrated to drive EV release (Van Meteren et al. 2019).

The recapitulation of the reduced abundance of distinct miRNAs in EVs as previously demonstrated in vivo by IS and high shear stress in vitro, further suggests that the combination of high shear stress and uraemia may, at least in part, be responsible for the release of EC‐EV specifically in HD patients and contribute to an altered functional EV profile in these patients. Notably, previous studies have suggested IS—through activation of the AhR—as a driver of impaired angiogenesis (Arinze et al. 2022; Salyers et al. 2021). Therefore, it can be speculated that EC‐EVs may provide a mechanistic link between IS and impaired angiogenesis. Consistent with such a sequence of events, in which IS‐mediated activation of the AhR drives the release of EVs with deleterious properties for the vasculature, IS has recently been demonstrated to induce anti‐angiogenic EV properties in endothelial cells (Zietzer et al. 2022). We also tested whether similar effects would be found in other cell types. However, our data suggest that we have uncovered an endothelial cell‐specific mechanism and that further studies are needed to elucidate the mechanisms of advanced CKD effects on EV release and cargo of hematopoietic cell lines.

Longitudinally, we show that KTx reverses altered endothelial EV release and normalizes EV miRNA content and downstream transcriptomic changes in angiogenesis‐related genes. Consequently, EC‐EVs may not only be biomarkers of established CVD, but rather reflect the current vascular disease process, as KTx patients do not show CVD reversal, but a lower CVD risk compared to patients remaining on dialysis (Rao and Coates 2018; Grabitz et al. 2024). This risk reduction is associated with a normalization of EC‐EVs in KTx recipients, which may thus serve as a particularly valuable biomarker in longitudinal studies. Notwithstanding, confounding factors in patients receiving transplantation, including immunosuppression need to be considered in future studies, as for example mTOR inhibition was previously shown to impact EV release (Gao et al. 2020). In the present study, none of the patients received mTOR inhibitors and to the best of our knowledge, there is no direct evidence for effects of other immunosuppressive drugs on EV release at present.

There are several limitations to this study. The group size of the clinical study was limited, because it focused specifically on children with a low prevalence of CKD. Nevertheless, we were able to also enrol patients after KTx, allowing comparisons of CKD patients also with KTx recipients as another collective with sufficient kidney function, and adding intra‐individual comparisons of CKD patients after restoring kidney function. Nonetheless, future studies should be performed on a larger scale to verify the present findings and to investigate possible effects of underlying kidney disease on plasma EV characteristics. Furthermore, it remains to be elucidated in future studies which cells are responsible for the altered EV cargo in CKD. While our data on altered EV miRNA cargo in EC‐EVs upon shear stress and IS exposure in vitro mimicked the plasma EV phenotype in dialysis patients, the limited biomaterial availability in our paediatric study made it impossible to sort for specific EV populations and subsequently analyse the cargo of individual EV subpopulations. Additionally, we did not specifically test the functional relevance of specific miRNAs for angiogenesis, but we were able to show that target genes of dysregulated miRNAs in CKD EVs are increased in endothelial cells upon exposure to CKD EVs versus healthy/KTx EVs.

In conclusion, we show that children with advanced CKD, particularly those on HD, have a phenotype of increased endothelial EV release, coupled with dysregulated EV miRNA cargo and the ability to impair angiogenesis. Alterations in EV formation and cargo may be driven, at least in part, by uremic TRP metabolites and vascular mechanical stress. High vascular shear stress is not only present in HD patients with AVF but is also found at atherosclerotic lesions in other patients with severe CVD (Slager et al. 2005). One could therefore speculate that the combination of uraemia and high shear stress, for example at atherosclerotic plaques, may also drive the altered EV release observed in adult CKD patients not yet on dialysis. We identify several EV miRNAs as promising therapeutic targets that could be tested in future studies to restore impaired angiogenesis in CKD, for example, by RNA interference via administration of nanoparticle‐bound small interfering RNAs mimicking miRNA sequences, as recently shown to be effective in hereditary transthyretin amyloidosis (Adams et al. 2018). Our study provides further insights into the diverse mechanisms of CVD development in CKD. Advances in the field of EVs and circulating miRNAs may thus contribute to a better understanding of vascular pathophysiology and ultimately reduce the burden of CVD in CKD.

## Author Contributions

Felix Behrens, Johannes Holle, Wolfgang M. Kuebler and Szandor Simmons conceived the study. Felix Behrens, Johannes Holle, István A. Szijártó, Uwe Querfeld, Philip Bufler, Julia Thumfahrt, Dominik Müller and Nicola Wilck performed and oversaw the clinical study. Felix Behrens, Laura F. Ginsbach, Benjamin C. Krause, Ulrike Bruning, Fabian L. Kriegel, Toralf Kaiser, Katrin Lehmann, Fabian Schumacher, Dörte Lodka, Carina Hoffmann, André A. Borchardt, Lisa Peters, Laura Michalick, Andreas Luch, Burkhard Kleuser, Jennifer A. Kirwan, Mir‐Farzin Mashreghi, Wolfgang M. Kuebler and Szandor Simmons performed and oversaw experiments. Felix Behrens, Johannes Holle, Chia‐Yu Chen, Harithaa Anandakumar, Pawel Durek, Frederick F. Heinrich, Sofia K. Forslund, Mir‐Farzin Mashreghi, Ulrike Löber, Hendrik Bartolomaeus, Wolfgang M. Kuebler and Szandor Simmons analysed the data. Felix Behrens, Johannes Holle, Hendrik Bartolomaeus, Wolfgang M. Kuebler and Szandor Simmons drafted and revised the manuscript. All authors read and approved the manuscript for publication.

## Conflicts of Interest

The authors declare no conflicts of interest.

## Supporting information



Supporting information

## Data Availability

Original data from patient EV small RNA sequencing will not be made publicly available, as potential single nucleotide polymorphisms raise data confidentiality and privacy concerns. Upon reasonable request, they will be made available by the corresponding author after approval by the local ethics committee. Supporting original HAoEC bulk RNA sequencing data are available from the NCBI Sequence Read Archive (bioproject accession number PRJNA1104603, https://www.ncbi.nlm.nih.gov/bioproject/1104603). Original metabolomics data are available from MetaboLights (Haug et al. 2019) (accession number MTBLS5075, https://www.ebi.ac.uk/metabolights/MTBLS5075). All analysis codes are available at https://github.com/CCY‐dev/EV_paper_code/
