# Supplementary material for: Circulating Extracellular Vesicles as Putative Mediators of Cardiovascular Disease in Paediatric Chronic Kidney Disease
Source: J Extracell Vesicles. 2025 Mar 21;14(3):e70062. doi: 10.1002/jev2.70062 (PMC11926757; doi:10.1002/jev2.70062)
Supplement: Supplementary file 1 — Supporting information [file JEV2-14-e70062-s001.pdf]

Supplement to: Circulating extracellular vesicles as putative mediators of cardiovascular disease in pediatric chronic kidney disease

Behrens*, Holle* *et al.*

**Table of contents**		**page**
**Supplemental materials and methods**		2
**Supplemental figures**		
Figure S1	Study design	16
Figure S2	Plasma EV western blot	17
Figure S3	Representative images plasma EV flow cytometry	18
Figure S4	Quantification of plasma EV flow cytometry	19
Figure S5	Plasma EV sphingolipidomics	20
Figure S6	Plasma EV miRNA sequencing	21
Figure S7	miRNA target identification	22
Figure S8	HAoEC DEG – miRNA target gene matches	23
Figure S9	Vascular smooth muscle cell proliferation upon CKD EV exposure	24
Figure S10	Individual tryptophan plasma metabolites I	25
Figure S11	Individual tryptophan plasma metabolites II	26
Figure S12	HAoEC EV release after exposure to uremic toxins	27
Figure S13	Platelet and macrophage EV release and miRNA cargo after exposure to indoxyl sulfate	28
**Supplemental tables**		
Table S1	MiFlowCyt-EV guideline report	26
Table S2	Flow cytometry antibody list	27
Table S3	List of tryptophan metabolites	28
Table S4	Tryptophan metabolite – EV correlation analyses	29
Table S5	Baseline clinical characteristics of the hemodialysis cohort	30
**References**		31

Supplemental materials and methods

Transmission electron microscopy (TEM)

EVs for TEM imaging were purified from 500 µL citrated plasma using the exoEasy Maxi Kit (Qiagen, Hilden, Germany) according to the manufacturer's protocols. EV eluates were then concentrated to 100 µL using Sartorius Vivaspin 2 columns with a 100kD molecular weight cut-off (Sartorius, Göttingen, Germany) and fixed in 2% paraformaldehyde in PBS. For negative staining, 15 µL of fixed eluates were applied to parafilm and carbon-coated EM grids were placed on top for 15 minutes at room temperature. The grids were then blotted onto moist filter paper (*aqua dest.*) and washed three times with *aqua dest*. Contrasting was achieved by placing the grids in 20 µL of 1% uranyl acetate for 20 seconds, and then the grids were again blotted on moist filter paper (*aqua dest.*) and air-dried. TEM was performed with a Leo EM 906 (Carl Zeiss, Oberkochen, Germany) at 27,800× magnification.

Immunoblotting

Immunoblotting for CD9, CD41 and GAPDH was performed on EVs isolated from 100 µL citrated plasma using the exoEasy Maxi Kit (Qiagen) according to the manufacturer's protocol. Protein was extracted by addition of 4× non-reducing SDS Laemmli buffer and incubation at 95°C for five minutes, and protein concentration was determined by bicinchoninic acid assay (BCA assay). 20 µg of protein and 5 µL of PageRuler prestained protein ladder (Thermo Fisher Scientific, Waltham, MA, USA) were loaded onto 1.5 mm 10% Bis-Tris gels. Protein was transferred to a 0.2 µm PVDF membrane and blocked with 5% non-fat milk for one hour. After three washes in TBS-T, the membrane was incubated overnight with primary antibodies in 5% BSA (CD9 (D8O1A) rabbit mAb, Integrin α2b (D8V7H) rabbit mAb (CD41), both 1:1,000, both Cell Signaling Technology, Cambridge, UK; GAPDH (0411) mouse mAb, 1:1,000, Santa Cruz Biotechnology, Dallas, TX, USA). After three washes in TBS-T the membrane was incubated for two hours with secondary antibodies in 5% BSA (donkey anti-rabbit-HRP polyclonal (NA 934), 1:2,000, GE Healthcare, Chicago, IL, USA, or rabbit anti-mouse-HRP polyclonal (P0260), 1:2,000, Agilent Technologies, Santa Clara, CA, USA). After three washes in TBS-T, the membrane was incubated for two minutes with HRP substrate (SuperSignal West Femto Maximum Sensitivity Substrat, Thermo Fisher Scientific) and imaged. The original images were processed in Adobe Photoshop by modifying the graduation curves for better visualization.

Nanoparticle Tracking Analysis (NTA)

EVs for NTA were purified by applying 50 µL of citrated plasma to Izon qEVoriginal 70nm size exclusion chromatography (SEC) columns (Izon Science, Christchurch, New Zealand) and the EV fraction was collected in 1.5mL of PBS according to the manufacturer's protocols. NTA measurements were performed using a NanoSight LM20 (NanoSight, Amesbury, UK) equipped with a 632nm laser. SEC eluates were injected into the sample chamber using sterile syringes at room temperature and were analyzed in five positions for 60 seconds per position. NTA 3.0 software (NanoSight) was used for measurements and analysis. Raw concentration data were multiplied by 30 to calculate plasma concentrations. Statistical tests were performed with GraphPad Prism (GraphPad Software, San Diego, CA USA). Kruskal-Wallis test and Dunn's post hoc test were performed for cross-sectional data and Wilcoxon test for longitudinal data, $p < 0.05$ was considered statistically significant.

Patient EV flow cytometry

4 µL of citrated plasma per patient per staining was diluted 1:50 in Annexin V binding buffer (BioLegend, San Diego, CA, USA). Four separate stainings were performed for each patient sample, all containing 1:4,000 FITC Annexin V (BioLegend) and two antibodies per staining (antibodies directed against i) CD41 (BV510, clone HIP8, 1:100, BioLegend) + CD235a (PerCP/Cy5.5, clone HI264, 1:100, BioLegend), ii) CD14 (BV421, clone 63D3, 1:100, BioLegend) + CD31 (BV711, clone WM59, 1:100, BioLegend), iii) CD3 (BV421, clone OKT3, 1:100, BioLegend) + CD20 (BV605, clone 2H7, 1:100, BioLegend), iv) CD66b (BV421, clone G10F5, 1:100, BD Biosciences, Franklin Lakes, NJ, USA) + CD68 (BV785, clone Y1/82A, 1:400, BioLegend), MiFlowCyt-EV guideline report in Table S1, full antibody list in Table S2). Annexin V staining was used as an internal control, but EVs were quantified independently of Annexin V staining. To quantify EVs, 50 µL of counting beads were added per sample (pediatric cohort: CountBright Absolute Counting Beads (Thermo Fisher Scientific); adult cohort: Precision Count Beads (Biolegend)). Samples were run on a BD Influx Cell Sorter (BD Biosciences) equipped with a 200mW 488nm laser, a 45mW 405nm laser and dedicated small particle optics. FlowJo v10.7 (FlowJo, Ashland, OR, USA) was used for data analysis. Statistical tests were performed with GraphPad Prism. Kruskal-Wallis test and Dunn's post hoc test were performed for cross-sectional data and Wilcoxon test for longitudinal data, $p < 0.05$ was considered statistically significant.

Plasma EV sphingolipidomics

EVs from 1 mL of patient plasma were isolated using the exoEasy Maxi Kit (Qiagen) according to the manufacturer's instructions and eluted in 400 µL elution buffer. They were then snap-frozen in liquid nitrogen and stored at -80°C. 40 µL EV suspensions were subjected to lipid extraction using 1.5 mL methanol/chloroform (2:1, v:v) as previously described[1]. The extraction solvent contained C17 ceramide (C17 Cer) and d_{31}-C16 sphingomyelin (d_{31}-C16 SM) (both Avanti Polar Lipids, Alabaster, USA) as internal standards. Chromatographic separations were performed on a 1290 Infinity II HPLC (Agilent Technologies, Waldbronn, Germany) equipped with a Poroshell 120 EC-C8 column (3.0 × 150 mm, 2.7 µm; Agilent Technologies). MS/MS analyses were performed using a 6495C triple-quadrupole mass spectrometer (Agilent Technologies) operating in the positive electrospray ionization mode (ESI+). Cer and SM were quantified by multiple reaction monitoring (qualifier product ions in parentheses): $[M\text{-}H_2O+H]^+ \rightarrow m/z$ 264.3 (282.3) for all Cer and $[M+H]^+ \rightarrow m/z$ 184.1 (86.1) for all SM subspecies (C16, C18, C20, C22, C24 and C24:1)[2]. Peak areas of Cer and SM subspecies, as determined by MassHunter Quantitative Analysis software (version 10.1, Agilent Technologies), were normalized to those of the internal standards (C17 Cer or d_{31}-C16 SM) followed by external calibration in the range of 1 fmol to 50 pmol on the column. The amounts sphingolipids determined were normalized to the actual protein content (as determined by the Bradford assay) of the EV suspension used for extraction.

Lipidomics data analysis

The differential abundance of sphingolipids between patient groups was analyzed using the Wilcoxon rank-sum test to calculate p-values, Benjamini-Hochberg FDR (BH-FDR), FDR-corrected p-values < 0.1 were considered statistically significant, while the effect sizes (Cliff's delta) were calculated using the dmes() function in the orddom R package[3].

Principal component analysis (PCA) of the lipidomics data was performed and visualized using the factoextra R package[4].

Plasma EV small RNA sequencing

500 µL of citrated plasma per patient was thawed and EV RNA was isolated using the ExoRNeasy Midi Kit (Qiagen) according to the manufacturer's protocol. In brief, plasma was passed through the columns to bind EVs to the membrane, EVs were the lysed , RNA was extracted using Qiazol reagent, purified using RNeasy MinElute columns, and dissolved in 12 µL of RNase-free water. 7 µL of RNA was used to generate cDNA libraries for small RNA

sequencing using the SMARTer smRNA-Seq Kit for Illumina (Takara Bio USA, San Jose, CA, USA). The final libraries were quality-checked for base pair length (~172bp) using a Fragment Analyzer (Advanced Analytical Technologies, Heidelberg, Germany) and cDNA content was measured using the Qubit dsDNA HS Assay Kit (Thermo Fisher Scientific). Sequencing was performed on a MiSeq sequencer using the MiSeq Reagent Kit v3 (150-cycle, both Illumina, San Diego, CA, USA).

Small RNA sequencing data analysis

MicroRNA (miRNA) reads were mapped using miRDeep2[5]. Differential expression of miRNAs between patient groups was analyzed using DESeq2[6]. In brief, raw miRNA data were converted into a DESeqDataSet using the function DESeqDataSetFromMatrix() and then pairwise comparisons between patient groups were performed using the function lfcShrink(type = "ashr"). P-values were corrected by BH-FDR. FDR-corrected p-values < 0.1 were considered significant. In addition to DESeq2, the results of the DESeq2 miRNA analysis were further confirmed using an adaptation of the LongDat R script[7]. To adapt to the cross-sectional analysis used here (group-to-group comparison), the effect size calculation script was modified to calculate the between-group effect size. Here, the "count" mode of LongDat was used, which means that the data were fitted with negative binomial models. MiRNAs with model p-values (FDR-corrected) < 0.1 and post-hoc test p-values (FDR-corrected) < 0.1 were considered significant.

Partial Least Squares Discriminant Analysis (PLS-DA) of the miRNAs was performed using the caret R package[8] and plotted using the ggplot2 package[9].

Identification of miRNA targets

Target genes of all 31 miRNAs that were significantly altered in CKD, PD and/or HD patients compared to healthy donors and/or KTx patients according to DESeq2 analyses and confirmed by LongDat were identified using TargetScanHuman (v8.0, targetscan.org)[10]. Target genes of all 31 miRNAs were pooled and filtered for miRNA-gene matches with a cumulative weighted context++ score below a cut-off of –0.3 to exclude matches with low gene regulation probability. Gene set enrichment analysis was then performed using the PANTHER overrepresentation test (PANTHER 17.0)[11,12] via geneontology.org. The reference list was set to "Homo sapiens" and Fisher's exact test with FDR correction was applied, p-values (FDR-corrected) < 0.05 were considered statistically significant. Only the most specific gene ontology

(GO) terms from hierarchical trees of significantly altered GO terms and GO terms with fold enrichment ≥ 1.5 or ≤ 0.5 were used for graphical illustration and further analysis. To trace back the influence of specific miRNAs on the identified GO terms the database of miRNA-gene matches with a cumulative weighted context++ score < –0.3 was filtered for the target genes that matched for the selected GO terms with fold enrichment ≥ 1.5, and the relative contribution of each of the 31 miRNAs to all miRNA-gene matches for each of the selected GO terms was calculated.

Patient EV miRNA RT-qPCR

The five miRNAs that were significantly altered in CKD EVs according to small RNA sequencing and had the highest predicted contribution to the enriched GO terms in the miRNA target analysis were validated by RT-qPCR in the same patient samples that were used for sequencing (hsa-miR-19a-3p, hsa-miR-142-3p, hsa-miR-103a-3p, hsa-let-7d-5p, hsa-miR-24-3p). In addition, the single miRNA found to be upregulated in dialysis patients was also assessed by qPCR (hsa-miR-4485-3p). cDNA was generated from 2 µL of RNA using the TaqMan Advanced miRNA cDNA Synthesis Kit (Thermo Fisher Scientific) according to the manufacturer's instructions. RiboLock RNase inhibitor (Thermo Fisher Scientific) was added before starting the reactions. Taqman Advanced miRNA Assays were used for qPCR (Thermo Fisher Scientific) according to the manufacturer's instructions. In brief, 5 µL of cDNA was diluted 1:10 in 0.1X trypsin-EDTA buffer. 5 µL of diluted cDNA was added to 15 µL of qPCR master mix containing 10 µL TaqMan Fast Advanced Master Mix (2X), 1 µL of the miRNA-specific TaqMan Advanced miRNA Assay (20×) (both Thermo Fisher Scientific) and 4 µL RNase-free water. qPCR reactions were performed in duplicates on a QuantStudio 7 Flex Real-Time PCR System (Thermo Fisher Scientific) according to manufacturer's instructions (40 cycles). Statistical testing was performed with GraphPad Prism. Normal distribution of data was tested using the Kolmogorov-Smirnov test. Kruskal-Wallis test and Dunn's post hoc test for non-normally distributed data or one-way ANOVA and Sidak's post hoc test for normally distributed data were performed as appropriate for cross-sectional data, and Wilcoxon or paired Student's *t*-test for longitudinal data. $P < 0.05$ was considered statistically significant.

Treatment of HAoECs with CKD EVs and HAoEC transcriptomics

To assess the effect of CKD EVs on the vasculature HAoECs were treated with EVs from healthy children and pediatric CKD patients undergoing hemodialysis or after kidney transplantation. EVs were then isolated from 1 mL of patient plasma using the exoEasy Maxi

Kit (Qiagen) according to the manufacturer's instructions and eluted in 400 µL of elution buffer. The samples were then snap-frozen in liquid nitrogen and stored at -80°C.

Commercially available Human Aortic Endothelial Cells (HAoECs) were cultured in Endothelial Cell Growth Medium MV with the appropriate SupplementMix (all PromoCell, Heidelberg, Germany) and 1% penicillin/streptomycin. HAoECs were seeded at confluence in a gelatin-coated 24-well plate and incubated overnight at 37°C overnight to allow attachment. The cells were then washed with PBS and incubated for 18 hours at 37°C in duplicates with 40 µL of EVs or vehicle control (buffer XE from the exoEasy Maxi Kit (Qiagen)) diluted 1:10 in basal medium (Endothelial Cell Growth Medium MV (PromoCell)).

After incubation, the medium was removed and Qiazol (Qiagen) chloroform isolation followed by purification with components of the exoRNeasy Midi Kit (Qiagen). 500 µL Qiazol was added per well, and the cells were scraped from the bottom of the well. The lysate was mixed with 100 µL of chloroform and then centrifuged at 12,000×g for 15 min at 4°C. The upper aqueous phase was collected and duplicates were pooled. Two volumes of ethanol were added and the mixtures were transferred to RNeasy MinElute spin columns and centrifuged at 12,000 rpm for 15 seconds at room temperature. The columns were washed once with RWT buffer and twice with RPE buffer before drying the columns by centrifugation with the lid open at 14,000 rpm for five minutes at room temperature and eluting the RNA in 12 µL RNase-free water by centrifugation at 14,000 rpm for one minute. RNA was stored at -80°C. RNA concentration and quality were analyzed using a NanoDrop spectrophotometer (Thermo Fisher Scientific) and the RNA Quality Number was measured using a fragment analyzer (Advanced Analytical Technologies).

cDNA was generated from 10 ng RNA using the SMART-Seq v4 Ultra Low Input RNA Kit for Sequencing (Takara Bio USA) and libraries were prepared from 1 ng cDNA using the Nextera XT Library Prep Kit (Illumina). cDNA and library quality, DNA concentration and fragment size were measured using a fragment analyzer (Advanced Analytical Technologies) and the Qubit dsDNA HS Assay Kit (Thermo Fisher Scientific). Sequencing was performed on a NextSeq 2000 using the NextSeq 2000 P3 reagents (100 cycles, both Illumina).

HAoEC bulk RNA sequencing data analysis

RNA reads were mapped using STAR[13] and gene level quantification was performed using featureCounts[14]. Read counts were imported into R and batch-corrected between sequencing

rounds using the ComBat function of the sva package[15]. Differential expression analysis was performed using DESeq2[6]. Size factors were estimated to account for differences in library size, dispersion was estimated using the apeglm method, and differential testing was performed using shrinkage estimators. Results were extracted using custom contrasts comparing HD EV to healthy donor and KTx EV treatment. Subsequent analyses were performed only on genes belonging to Gene Ontology (GO) terms identified by prior miRNA sequencing and potentially involved in vascular function: cellular response to platelet-derived growth factor stimulus (GO:0036120), G1/S transition of the mitotic cell cycle (GO:0000082), regulation of smooth muscle cell proliferation (GO:0048660), regulation of angiogenesis (GO:0045765), negative regulation of cell migration (GO:0030336) and angiogenesis (GO:0001525). PCA was performed on the variance-stabilized transformed counts using the plotPCA function on the subset of genes. The heatmap was generated using the pheatmap package[16] and met the criteria of $P < 0.05$ and absolute log2 fold change > 1. To verify the matches of differentially enriched genes (DEGs) with miRNA target genes, the DEGs were cross-compared with the same list of miRNA target genes that was used for gene set enrichment. Matches of HAoEC DEGs with miRNA target genes were visualized in a circus plot using the circlize R package[17].

Vascular tube formation, endothelial migration and proliferation after exposure to CKD EVs

Human Umbilical Vein Endothelial Cells (HUVECs) were obtained shortly after isolation[18], cultured in Endothelial Cell Medium MV with the appropriate SupplementMix (all PromoCell, Heidelberg, Germany) and 1% penicillin/streptomycin, and used for experiments at passages 3-7. Plasma EVs were isolated using Exo-spin mini columns (Cell guidance systems, Cambridge, UK) with 100 µL citrated plasma that was eluted in 180 µL PBS. For vascular tube formation experiments confluent cells were starved in a T75 flask overnight in DMEM (Gibco, Grand Island, NY, USA) containing 2% FCS and 1% penicillin/streptomycin. 96-well plates were prepared by adding 50 µL Matrigel (Corning, Corning, NY, USA) containing 30 ng/mL vascular endothelial growth factor A (VEGF-A, Abcam, Cambridge, UK) per well and incubated at 37°C for 30 minutes. Subsequently, 40,000 cells per well were seeded on the polymerized Matrigel in 80 µL endothelial cell medium MV and 20 µL EVs from either healthy donors or HD patients were added in duplicate. Cells were incubated for 6 hours at 37°C and brightfield images were captured on an EVOS M5000 microscope (Thermo Fisher Scientific) at 10×. Vascular tube-like structures per field of view were counted (typical elongated character with maximum width of two cells) and *ImageJ Angiogenesis Analzyer* software[19] was used for automated analysis of vascular-like networks. Three images per well were analyzed and the

mean of both duplicates was used for subsequent statistical analyses. Analysis was performed on unaltered images; for representative images in the manuscript, grading curves were altered using Adobe Photoshop for better visualization.

To assess endothelial migration, 2-well culture inserts (Ibidi, Gräfelfing, Germany) were placed in a 24-well plate and 20,000 HUVECs were seeded on each side of the insert. The cells were allowed to attach for 12 hours and starved overnight in DMEM containing 2% FCS and 1% penicillin/streptomycin. The inserts were then removed and 400 μL Endothelial Cell Medium MV and 100 μL EVs from either healthy donors or HD patients were added. Brightfield microscopy was performed on an EVOS M5000 microscope at 10× immediately and after 4 hours of incubation at 37°C. Three images per well were analyzed for both time points and gap area was calculated using ImageJ. The relative reduction in gap area at 4 hours was used for statistical analysis. Analysis was performed on unaltered images; for representative images in the manuscript, grading curves were altered using Adobe Photoshop for better visualization.

For proliferation analyses 20,000 HUVECs were seeded per well in a 96-well plate and allowed to adhere for 12 hours. They were then incubated with 80 μL medium and 20 μL EVs from either healthy donors or HD patients for 18 hours in duplicates. For Ki-67 immunofluorescence, the cells were then washed three times with PBS, fixed with 4% PFA for 15 minutes and permeabilized with 0.25% Triton X-100. The cells were blocked with 3% BSA for 10 minutes. The cells were then washed three times with PBS-T and incubated with anti-Ki-67 antibody (rabbit anti-human, polyclonal, 1:400 in PBS-T, Abcam) for one hour. The cells were then washed three times with PBS-T and incubated with Alexa 568 anti-rabbit IgG (goat, polyclonal, 1:400 in PBS-T, Thermo Fisher Scientific) for one hour. The cells were washed three times with PBS-T and DAPI was added in PBS before subsequent fluorescence imaging on an EVOS M5000 microscope at 40×. DAPI^{+} and Ki-67^{+} nuclei were counted in three images per well, the Ki-67^{+} fraction was calculated and the mean of both duplicates was used for subsequent statistical analysis.

For all HUVEC assays statistical analyses were performed using the Mann-Whitney *U* test on GraphPad Prism, where $p < 0.05$ was considered statistically significant.

Vascular smooth muscle cell formation after exposure with CKD EVs

Human Aortic Smooth Muscle Cells (HAoSMCs, PromoCell) were cultured using Smooth Muscle Cell Growth Medium 2. To assess proliferation upon exposure to plasma EVs from HD

patients as compared to healthy donors, Ki-67 immunofluorescence was performed as described above for HUVECs, with two differences: cells were allowed to attach for 24 hours due to slower growth characteristics, and microscopic images were taken at 20× due to lower cell density. GraphPad Prism was used for statistical analysis, where $p < 0.05$ was considered statistically significant according to the Mann-Whitney *U* test.

Targeted tryptophan metabolomics

Plasma metabolomics was focused on tryptophan (TRP) metabolites and was performed as previously described[20] with an expansion of the analyzed metabolite panel to 34 metabolites (Table S3). In brief, liquid chromatography-mass spectrometry (LC-MS) was performed on a 1290 Infinity 2D HPLC system (Agilent Technologies, Santa Clara, CA, USA) combined with a TSQ Quantiva triple quadrupole mass spectrometer with a heated ESI source (Thermo Fisher Scientific) using 150 µL of EDTA plasma per patient. Data were exported to Skyline v.19.1 - 64 bit to identify and quantify peak intensity and area. 31 of 34 metabolites could be analyzed in quantitatively, for melatonin, formylanthranilate and picolonic acid only semi-quantitative measurements could be performed for technical reasons and these metabolites were excluded from further analysis.

Metabolomics and integrative data analysis

The differential abundance of metabolites between patient groups was analyzed using the Wilcoxon rank-sum test for p-value calculation, BH-FDR-corrected p-values < 0.1 were considered statistically significant, while the effect sizes (Cliff's delta) were calculated using the dmes() function in the orddom R package[3].

Principal component analysis (PCA) of the metabolomics data was performed and visualized using the factoextra R package[4].

Longitudinal analysis of metabolomics data in patients before and after kidney transplantation was performed using the longdat_disc(data_type = "measurement") function in the LongDat R package[7]. LongDat analyzes the longitudinal data using a covariate-sensitive approach. Here, the analysis took EVs and medical variables as covariates. The measurement mode first normalized the data and then fitted the data with linear mixed-effect models. Metabolites with model p-values (FDR-corrected) < 0.1 and post-hoc test p-values (FDR-corrected) < 0.05 were considered significant.

TRP metabolites were correlated with EC-EV plasma concentrations using Pearson correlation in GraphPad Prism. Correlations with $p < 0.05$ were considered statistically significant.

HAoEC EV release on uremic toxin stimulation

Potential effects of TRP-derived uremic toxins on endothelial EV release were tested *in vitro*. HAoECs were cultured as described above and seeded at confluence (0.7×10^5 cells/well) in gelatin-coated 24-well plates and incubated overnight at 37°C to attach. The cells were then washed with PBS and incubated with 1 µM carboxyfluorescein succinimidyl ester (CFSE, Thermo Fisher Scientific) in PBS for 10 minutes at room temperature. CFSE was removed and the cells were incubated in medium at 37°C for 24 hours. After washing with PBS, cells were incubated with indoxyl sulfate (IS), xanthurenic acid (XA) (both Sigma-Aldrich, St. Louis, MO, USA) or formylkynurenine (FKYN) (Biozol, Eching, Germany) in 100 nm-filtered basal medium at concentrations ranging from 0.1 µM to 1 mM for two (all toxins) and eight hours (IS, XA). Vehicle controls with DMSO (IS, XA) and 0.6 mM NaOH (FKYN) were performed as appropriate.

After incubation, the supernatant was collected and centrifuged at 600×g for 15 minutes to remove cells. The resulting supernatant was then centrifuged twice at 2,500×g for 15 minutes to remove debris. All centrifugation steps were perfromed at room temperature without breaks. The cell-free supernatant was then snap-frozen in liquid nitrogen and stored at -80°C.

HSaVEC EV release on shear stress and uremic toxin stimulation

EV release from the venous endothelium under venous or arterial shear stress and concomitant vehicle or IS treatment was tested *in vitro*. Commercially available Human Saphenous Vein Endothelial Cells (HSaVECs) were cultured in Endothelial Cell Growth Medium 2 supplemented with the SupplementMix 2 (all PromoCell) and 1% penicillin/streptomycin. HSaVECs were seeded at confluence (2.2×10^5 cells/well) onto pre-coated (attachment factor, incubated for 20 minutes at 37°C) µ-slides I 0.6 Luer (Ibidi) in 150 µL growth medium and incubated overnight at 37°C to attach. The cells were then washed twice with 100 µL PBS and then stained with 100 µL 1 µM CFSE (Thermo Fisher Scientific) in PBS for 10 minutes at room temperature. CFSE was removed and the cells were incubated in medium at 37°C for 24 hours.

The Ibidi pump system was used to mimic venous and arterial flow on HSaVECs. Cells were washed twice with 100 µL PBS and then cultured with 100 nm-filtered basal medium. 13 mL

of the medium was added to the flow system. Unidirectional laminar flow was applied at 1 dyn/cm^2 for venous flow and 10 dyn/cm^2 for arterial flow. Different perfusion sets were required to mimic venous and arterial flow in combination with the µ-slides I 0.6 Luer. For venous flow, the blue perfusion set was used according to the manufacturer's instructions while for arterial flow the red perfusion set was used. For venous flow, HSaVECs were exposed directly to 1 dyn/cm^2, whereas for arterial flow, the flow rates were gradually increased (30 minutes 2.5 dyn/cm^2, 30 minutes 5 dyn/cm^2, then 10 dyn/cm^2) to prevent the cells from being ruptured by immediate high shear stress. Both flow conditions were combined with vehicle control (DMSO) or IS treatment (50 µM). HSaVECs were incubated under these conditions for 18 hours.

After incubation, the supernatant was collected and centrifuged at 600×g for 15 minutes to remove cells. The supernatant obtained was then centrifuged twice at 2,500×g for 15 minutes to remove debris. All centrifugation steps were performed at room temperature without breaks. Given the large amount of medium and expected low EV concentrations, EVs were isolated and concentrated from the cell-free supernatant obtained by multiple centrifugations using the exoEasy Maxi Kit (Qiagen) according to the manufacturer's instructions. Isolated EVs were snap-frozen in liquid nitrogen and stored at -80°C.

Platelet EV release upon uremic toxin stimulation

For platelet isolation, 3.5 mL of whole blood was drawn from healthy donors in 3.2% trisodium citrate tubes. Informed written consent was obtained in accordance with the Declaration of Helsinki, and the study was approved by the Ethics Committee of Charité – Universitätsmedizin Berlin (EA2/162/17). Whole blood was centrifuged at 200×g for 15 minutes without breaks. Platelet-rich plasma was transferred to siliconized glass tubes containing 1 mL of 10% citrate-phosphate-dextrose solution containing adenine (CPDA, Sigma Aldrich) in PBS and centrifuged at 1,000×g for 15 minutes. After incubation at room temperature for 20 minutes, the supernatant/platelet-poor plasma was removed and the platelets were resuspended in 2 mL of 10% CPDA. 50 µL of platelets were transferred to a new siliconized glass tube and containing 950 µL of 10% CPDA. The platelets were then incubated with 50 µM IS or vehicle (DMSO) for 3 hours at 30°C on a shaking incubator at 100 rpm . After incubation, the tubes were centrifuged at 600×g for 15 minutes and the supernatant was collected. The supernatant was then centrifuged twice at 2,500×g for 15 minutes to remove debris. All centrifugation steps were performed at room temperature without breaks. The cell-free supernatant was snap-frozen in liquid nitrogen and stored at -80°C.

Macrophage EV release on uremic toxin stimulation

10 mL of whole blood was drawn from healthy donors into K3 EDTA tubes. Informed written consent was obtained in accordance with the Declaration of Helsinki, and the study was approved by the Ethics Committee of Charité – Universitätsmedizin Berlin(EA2/162/17). Whole blood was diluted in PBS with 2% FCS and layered on 15 mL Pancoll density gradient (PAN-Biotech, Aidenbach, Germany) in 50 mL SepMate tubes (StemCell Technologies, Vancouver, BC, Canada) and centrifuged at 1,200×g for 10 minutes at room temperature. Plasma and peripheral blood mononuclear cells (PBMCs) were transferred to a new 50 mL tube, filled to 50 mL with PBS containing 2% FCS and centrifuged at 400×g for 10 minutes at room temperature. The supernatant was removed and the cells were resuspended in 2 mL of erythrocyte lysis buffer (155 mM NH_4Cl, 10 mM $NaHCO_3$, 1 mM EDTA in water) and incubated at 37°C for 7 minutes. The tubes were then filled to 50 mL with PBS with 2% FCS and centrifuged at 400×g for 10 minutes at room temperature. The supernatant was removed and the cells were resuspended in 50 mL with PBS containing 2% FCS and centrifuged again at 400×g for 10 minutes at room temperature. The supernatant was removed and the cells were resuspended in with PBS containing 2% FCS and counted. The cells were centrifuged at 400×g for 10 minutes at room temperature, resuspended at 5×10^5 cells/ml in RPMI with 1% penicillin/streptomycin, 50 mM β-mercaptoethanol and 10 mM HEPES without FCS and 1×10^6 cells were seeded per well in 12-well plates. The cells were incubated for 2 hours at 37°C and then incubated for 6 days with RPMI containing 1% penicillin/streptomycin, 50 mM β-mercaptoethanol, 10 mM HEPES, 20% FCS and 100 ng/mL macrophage colony-stimulating factor (M-CSF, Miltenyi Biotec, Bergisch Gladbach, Germany) with one medium exchange after 3 days for macrophage differentiation. The cells were then washed with PBS and incubated with 1 mL RPMI containing 1% penicillin/streptomycin, 50 mM β-mercaptoethanol, 10 mM HEPES and 20% FCS with either 50 μM IS or vehicle (DMSO). After incubation, the supernatant was collected and centrifuged at 600×g for 15 minutes. The supernatant was collected and centrifuged twice at 2,500×g for 15 minutes to remove debris. All centrifugation steps were performed at room temperature without breaks. The cell-free supernatant was snap-frozen in liquid nitrogen and stored at -80°C.

HAoEC, platelet, macrophage and HSaVEC EV flow cytometry

40μL annexin binding buffer (5X, Thermo Fisher Scientific) was added to 100 μL purified HAoEC, platelet or macrophage supernatant/100 μL isolated EVs from HSaVECs. HAoEC and

HSaVEC EVs were stained with 1:200 PeCy7 Annexin V (BioLegend) in addition to CFSE staining. Plt- and Mac-EVs were stained with 1:100 anti-CD41 (BV510, clone HIP8, BioLegend) or 1:400 CD68 (BV785, clone Y1/82A, BioLegend) antibodies respectively and 1:4,000 FITC Annexin V (BioLegend). Annexin V staining was used as internal control, but EVs were quantified independently of Annexin V staining. 50 µL Precision Count Beads (Biolegend) were added per sample to quantify EVs. Samples were run on a BD Influx Cell Sorter (BD Biosciences) equipped with a 200mW 488nm laser, a 45mW 405nm laser and dedicated small particle optics. FlowJo v10.7 software was used for data analysis. Statistical tests were performed with GraphPad Prism. Kruskal-Wallis test and Dunn's post hoc test for unpaired data or Wilcoxon test for paired data were performed as appropriate, $p < 0.05$ was considered statistically significant.

HSaVEC, platelet and macrophage EV miRNA RT-qPCR

To identify a potential mechanism of differential EV miRNA release in CKD, HSaVECs EVs from the aforementioned different flow and uremic conditions and Plt- and Mac-EVs from uremic or control conditions were analyzed for their miRNA content by RT-qPCR. The same six miRNAs as in patient EVs were analyzed (hsa-miR-19a-3p, hsa-miR-142-3p, hsa-miR-103a-3p, hsa-let-7d-5p, hsa-miR-24-3p, hsa-miR-4485-3p), with the exception of hsa-miR-4485-3p in Plt- and Mac-EVs. HSaVEC EV RNA was extracted from isolated EVs using a combination of Qiazol (Qiagen) chloroform isolation followed by purification with components of the exoRNeasy Midi kit (Qiagen). 140 µL of EVs purified with the exoEasy Kit (Qiagen) from HSaVEC supernatant (representing 1/3 of the total EVs from the *in vitro* experiments) were mixed with 700 µL Qiazol, 140 µL chloroform was added and samples were then centrifuged at 12,000×g for 15 min at 4°C. The upper aqueous phase was removed, two volumes of ethanol were added and the mixtures were transferred to RNeasy MinElute spin columns and centrifuged at 12,000 rpm for 15 seconds at room temperature. The columns were washed once with RWT buffer and twice with RPE buffer before drying by centrifugation at 14,000 rpm for five minutes with the lid open at room temperature and eluting the RNA in 10 µL of RNase-free water by centrifugation at 14,000 rpm for one minute. Plt- and Mac-EV RNA was isolated from 300 µL supernatant using the ExoRNeasy Midi Kit (Qiagen) according to the manufacturer's protocol (see "Patient plasma EV RNA isolation") and was also eluted in 10 µL of RNase-free water. RNA was stored at -80°C.

cDNA generation from 2 µL of RNA and qPCR were performed in the same way as for patient samples using the TaqMan Advanced miRNA cDNA Synthesis Kit and Taqman Advanced miRNA Assays respectively (both Thermo Fisher Scientific, see details above). qPCR was run for 60 cycles in anticipation of lower RNA levels. Statistical tests were performed with GraphPad Prism. Kruskal-Wallis test and Dunn's post hoc test for unpaired data or Wilcoxon test for paired data were performed as appropriate, $p < 0.05$ was considered statistically significant.

Supplemental Figure 1

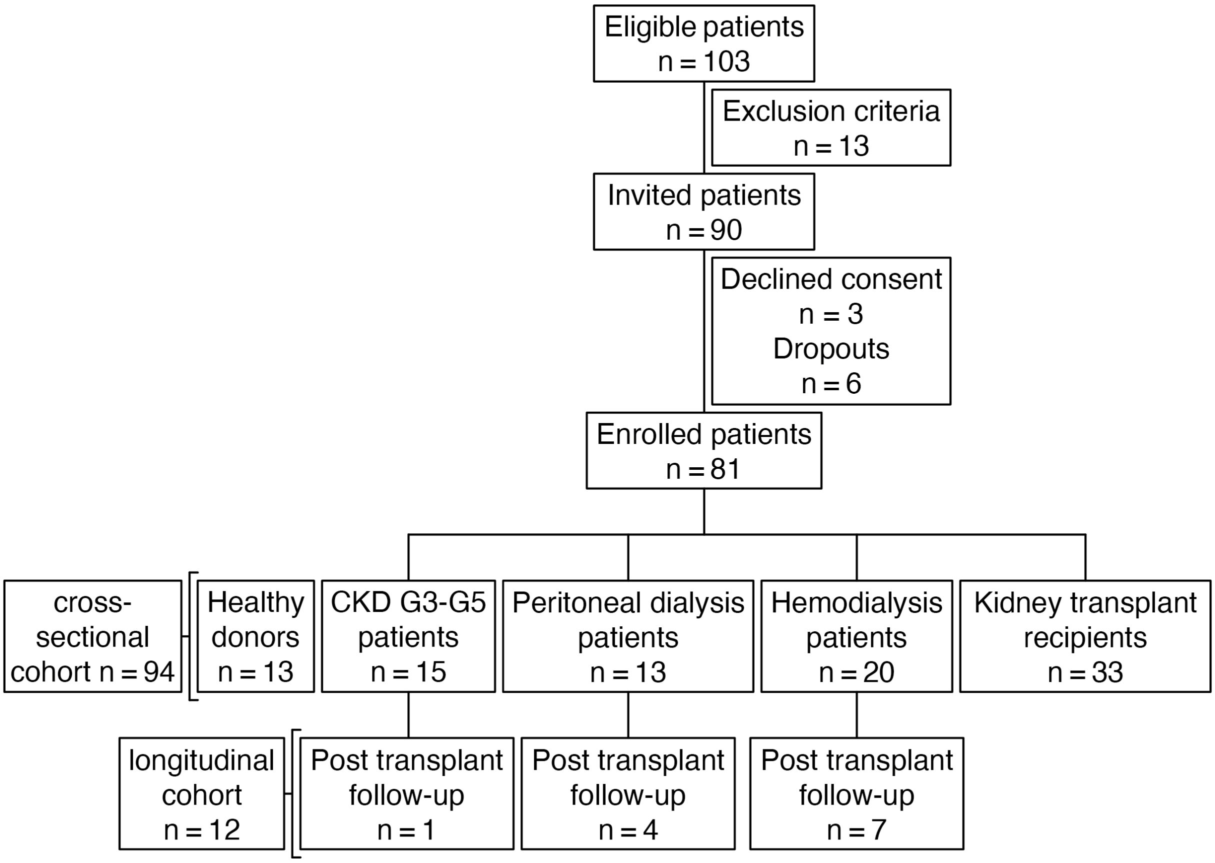

Figure S1. Study design pediatric CKD cohort. Of 103 eligible patients, 90 patients were invited to participate in this study (13 patients met the exclusion criteria). Three patients refused to participate and six patients dropped out due to loss to follow-up or difficulties with blood collection. In addition to the 81 CKD patients, 13 age-matched healthy donors with normal kidney function who were hospitalized for reasons other than kidney disease were enrolled, resulting in 94 patients in the cross-sectional cohort. During the study period twelve patients underwent successful kidney transplantation and were included in the longitudinal study with post-transplant follow-up.

Supplemental Figure 2

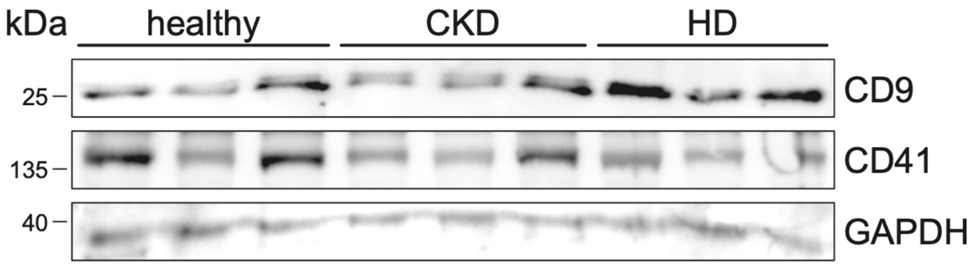

Figure S2. Plasma EV western blot. Expression of EV membrane protein CD9, platelet membrane protein CD41 and cytosolic/intravesicular protein GAPDH in EVs isolated from 100 µL of patient plasma from healthy donors, CKD patients without dialysis and patients on hemodialysis (HD).

Supplemental Figure 3

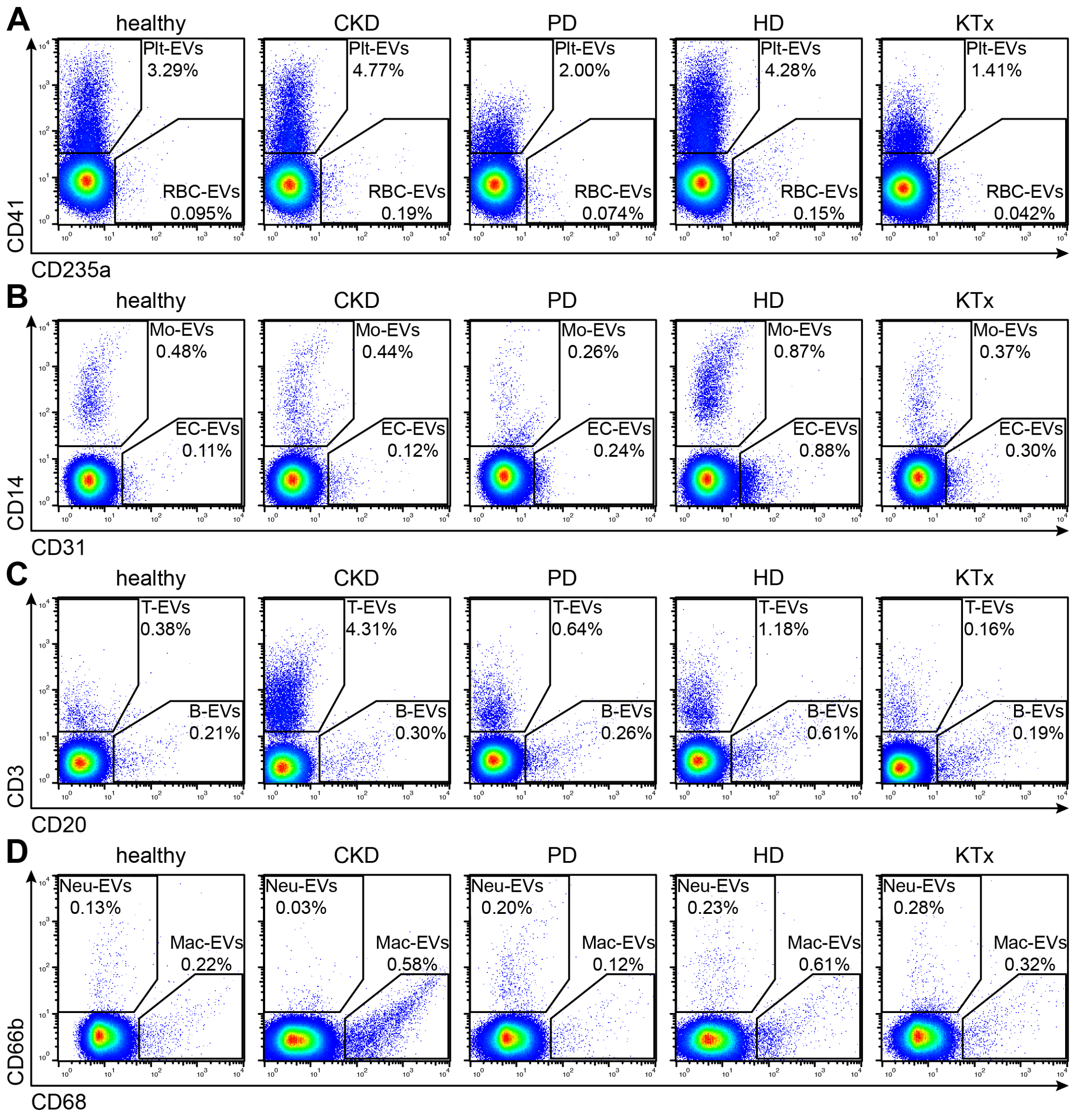

Figure S3. Representative images plasma EV flow cytometry. Pseudocolor plots showing the four plasma EV staining panels for all patient groups of the pediatric CKD cohort used to quantify **(A)** CD41$^+$ platelet-derived (Plt-) EVs and CD235a$^+$ red blood cell-derived (RBC-) EVs, **(B)** CD14$^+$ monocyte-derived (Mo-) EVs and CD31$^+$ endothelial (EC-) EVs, **(C)** CD3$^+$ T cell-derived (T-) EVs and CD20$^+$ B cell-derived (B-) EVs, and **(D)** CD66b$^+$ neutrophil-derived (Neu-) EVs and CD68$^+$ macrophage-derived (Mac-) EVs. HD hemodialysis patients, KTx kidney transplant recipients, PD peritoneal dialysis patients.

Supplemental Figure 4

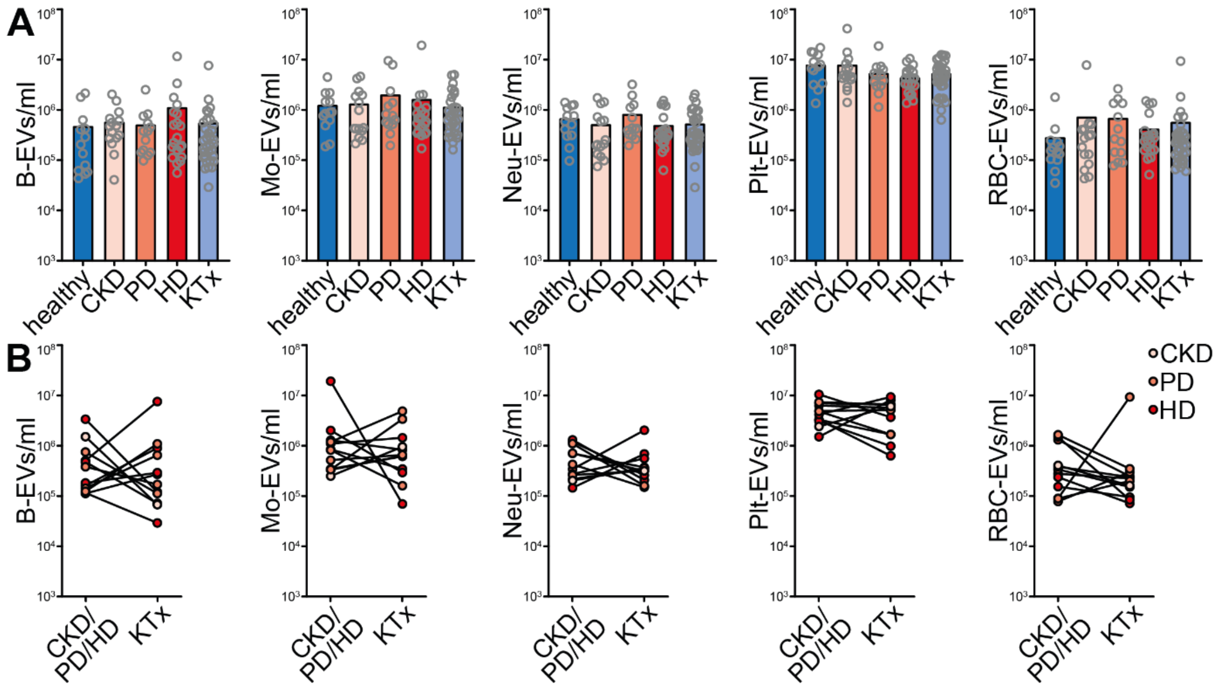

Figure S4. Quantification of plasma EV flow cytometry. Specialized multicolor EV flow cytometry was performed on plasma from all patients in the pediatric CKD cohort. Cross-sectional **(A)** and longitudinal **(B)** comparison of EVs of additional cellular origins: $CD20^+$ B cell-derived (B-) EVs, $CD14^+$ monocyte-derived (Mo-) EVs, $CD66b^+$ neutrophil-derived (Neu-) EVs, $CD41^+$ platelet-derived (Plt-) EVs and $CD235a^+$ red blood cell-derived (RBC-) EVs. HD hemodialysis patients, KTx kidney transplant recipients, PD peritoneal dialysis patients.

Supplemental Figure 5

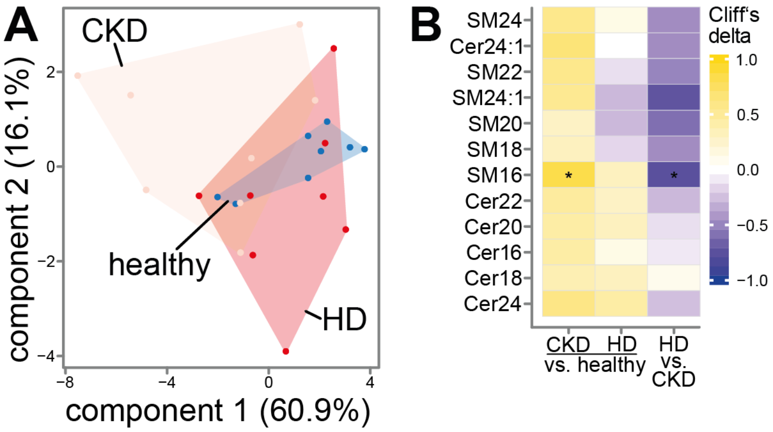

Figure S5. Plasma EV sphingolipidomics. Exploratory EV cargo analysis for sphingolipids (ceramides (Cer) and sphingomyelins (SM)) was performed in a subset of healthy donors, CKD patients without dialysis and hemodialysis (HD) patients (N=8 per group). Lipidomics was performed on isolated plasma EVs and sphingolipid concentrations were normalized to protein content. **(A)** Principal component analysis showed a large overlap between healthy donors and hemodialysis patients, whereas CKD patients without dialysis showed a partial separation. **(B)** This trend was confirmed at the metabolite level where CKD patients without dialysis showed different concentrations of total SM, SM16 and total ceramide as compared to healthy donors and HD patients. However, this trend was not consistent across different patient groups as evidenced by no significant differences between HD patients and healthy donors. **(A)** *$p < 0.1$ by Wilcoxon rank-sum test with FDR correction.

Supplemental Figure 6

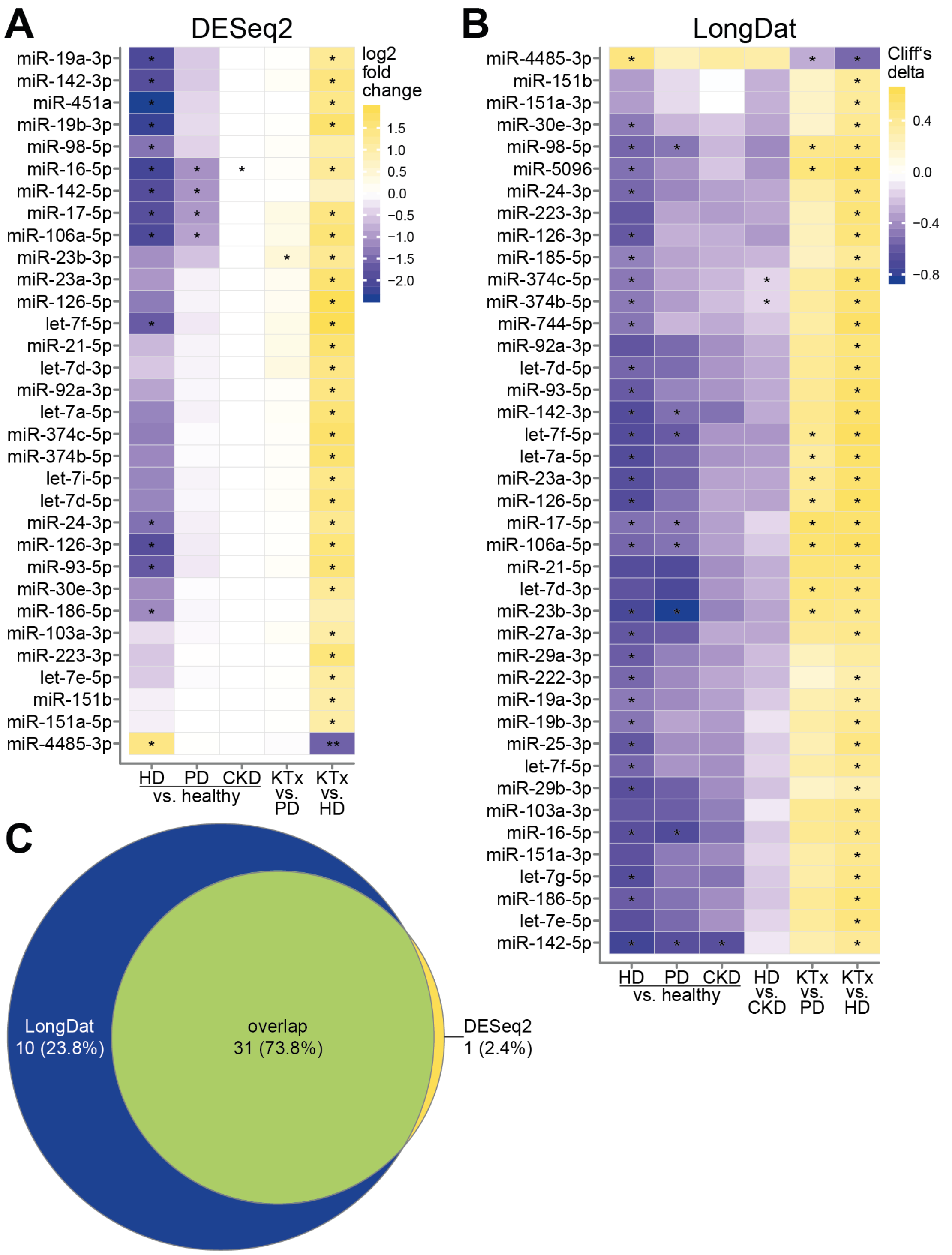

Figure S6. Plasma EV miRNA sequencing. Heatmaps showing significantly altered plasma EV miRNAs in CKD patients as analyzed by **(A)** DESeq2 and **(B)** LongDat**. (C)** Venn diagram comparing significantly altered miRNAs as identified by DESeq2 and LongDat. **p* (FDR-corrected) < 0.1. HD hemodialysis patients, KTx kidney transplant recipients, PD peritoneal dialysis patients.

Supplemental Figure 7

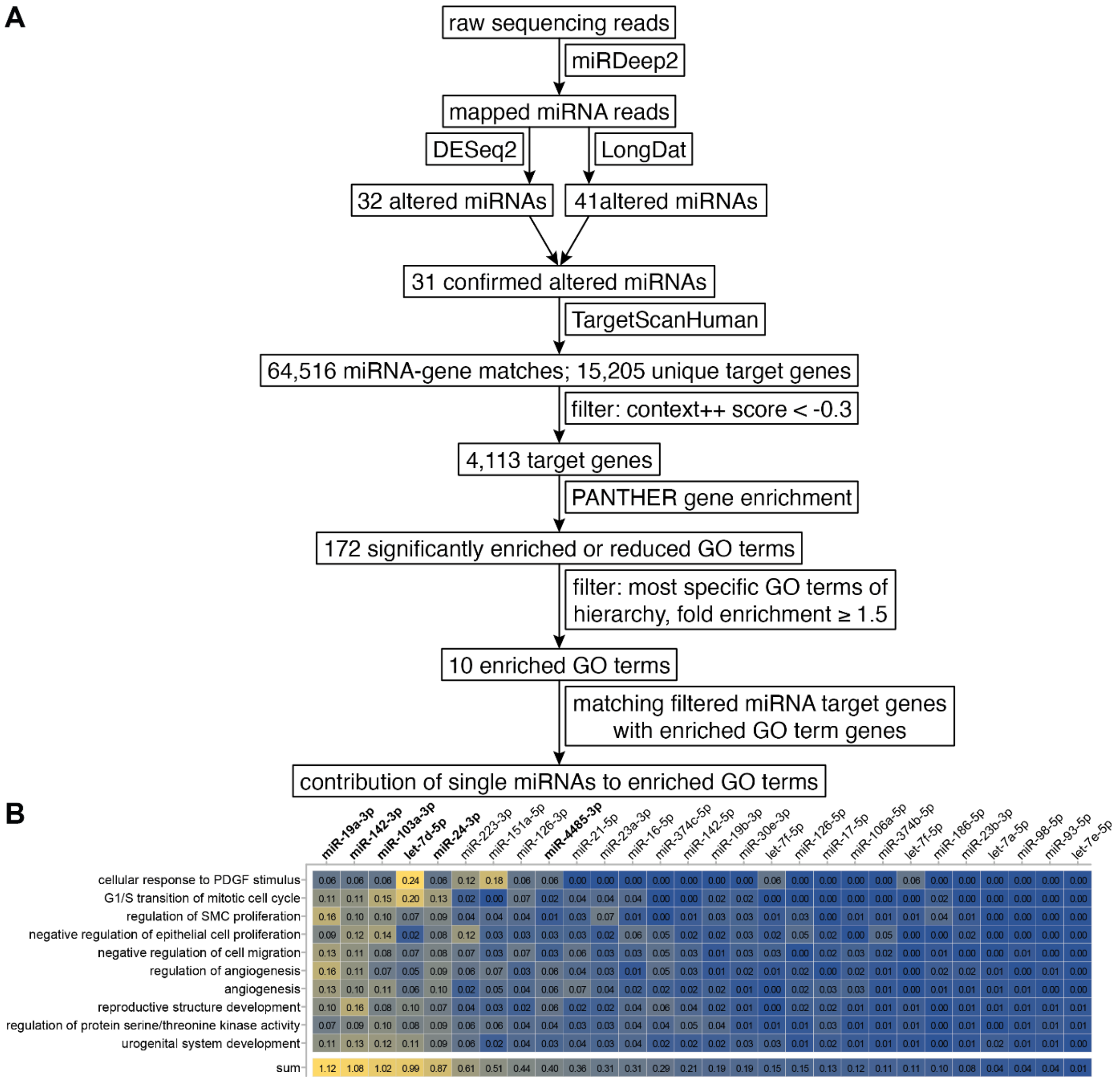

Figure S7. miRNA target identification. (A) Workflow for small RNA sequencing data analysis and miRNA target identification. **(B)** Heatmap of the relative contribution of individual miRNAs to enriched gene ontology (GO) terms, shown as the fraction of individual miRNA target gene matches for an enriched GO term, hence row sum = 1 for each GO term. Sums are shown as a surrogate for the global interference potential of individual miRNAs for the ten enriched GO terms. miRNAs selected for RT-qPCR validation are highlighted in **bold**.

Supplemental Figure 8

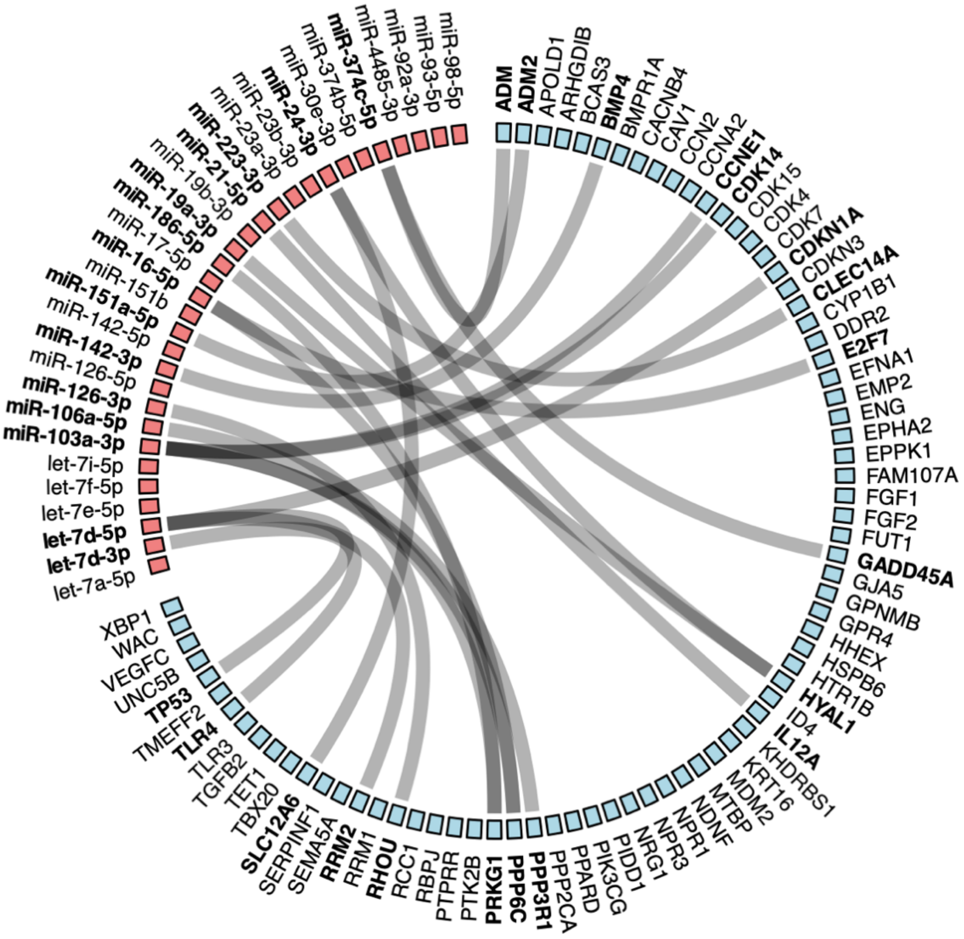

Figure S8. HAoEC DEG – miRNA target gene matches. Differentially enriched genes (DEGs) from Human Aortic Endothelial Cells (HAoECs) treated with healthy, hemodialysis or kidney transplant recipient plasma EVs (Figure 3B) were matched with predicted miRNA target genes from miRNAs differentially enriched in CKD EVs (as used for gene set enrichment analysis in Figure 3C). Matches are visualized in a circus plot, with matches indicated by connecting lines and bold font.

Supplemental Figure 9

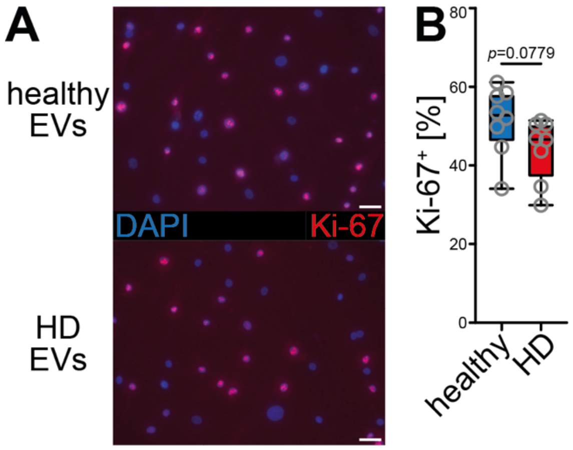

Figure S9. Vascular smooth muscle cell proliferation after exposure to CKD EVs. Human Aortic Smooth Muscle Cells (HAoSMCs) were exposed to EVs from either healthy donors or hemodialysis (HD) patients for 18 hours. HAoSMC proliferation was measured by immunofluorescence (Ki-67 expression). **(A)** Representative microscopic images of HAoSMCs after 18 hours of incubation with healthy/HD EVs. **(B)** Unaltered proliferation after exposure to HD EVs as compared to healthy EVs as measured by the proportion of Ki67^{+} nuclei. **(A)** Scale bar 50 µm (20×). **(B)** P-value according to Mann-Whitney *U* test. HD hemodialysis patients.

Supplemental Figure 10

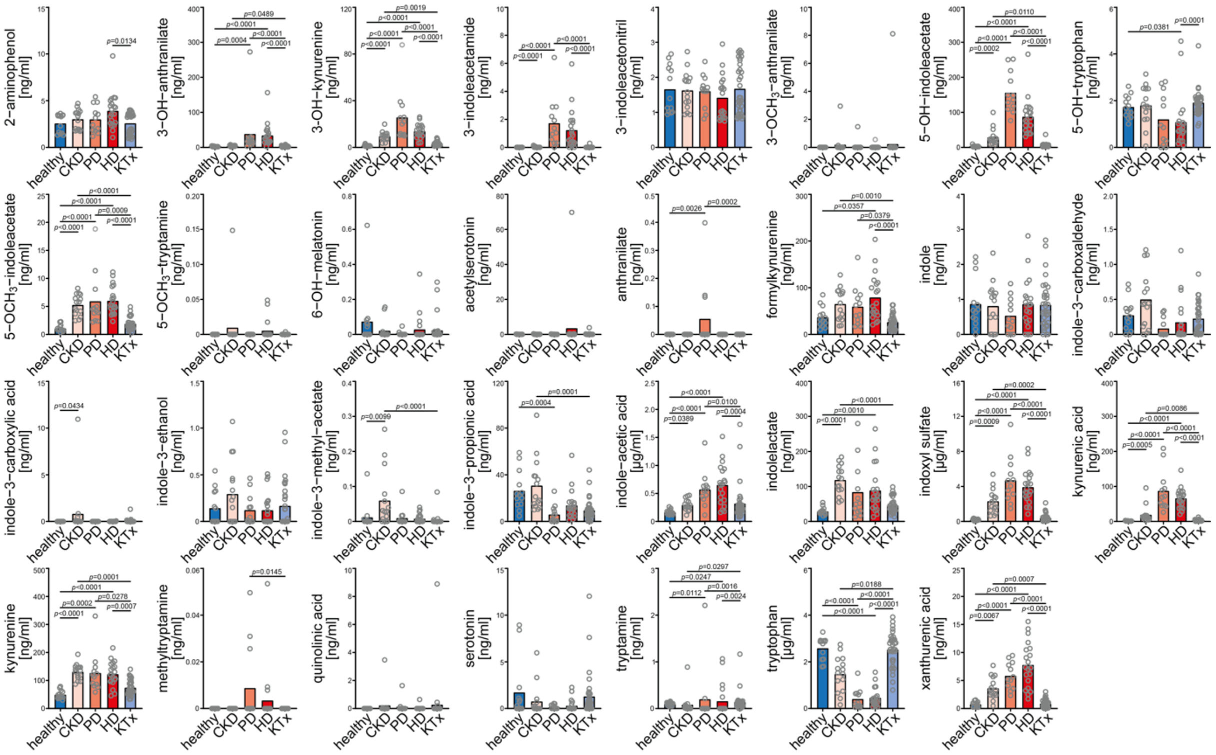

Figure S10. Individual tryptophan plasma metabolites I. Targeted plasma metabolomics was performed for 34 tryptophan metabolites of which 31 could be quantified in the pediatric CKD cohort. Bar graphs showing individual metabolite concentrations in cross-sectional group comparisons. Melatonin, formylanthranilate and picolonic acid could not be quantified for technical reasons. P-values according to Kruskal-Wallis test and Dunn's post hoc test. HD hemodialysis patients, KTx kidney transplant recipients, PD peritoneal dialysis patients.

Supplemental Figure 11

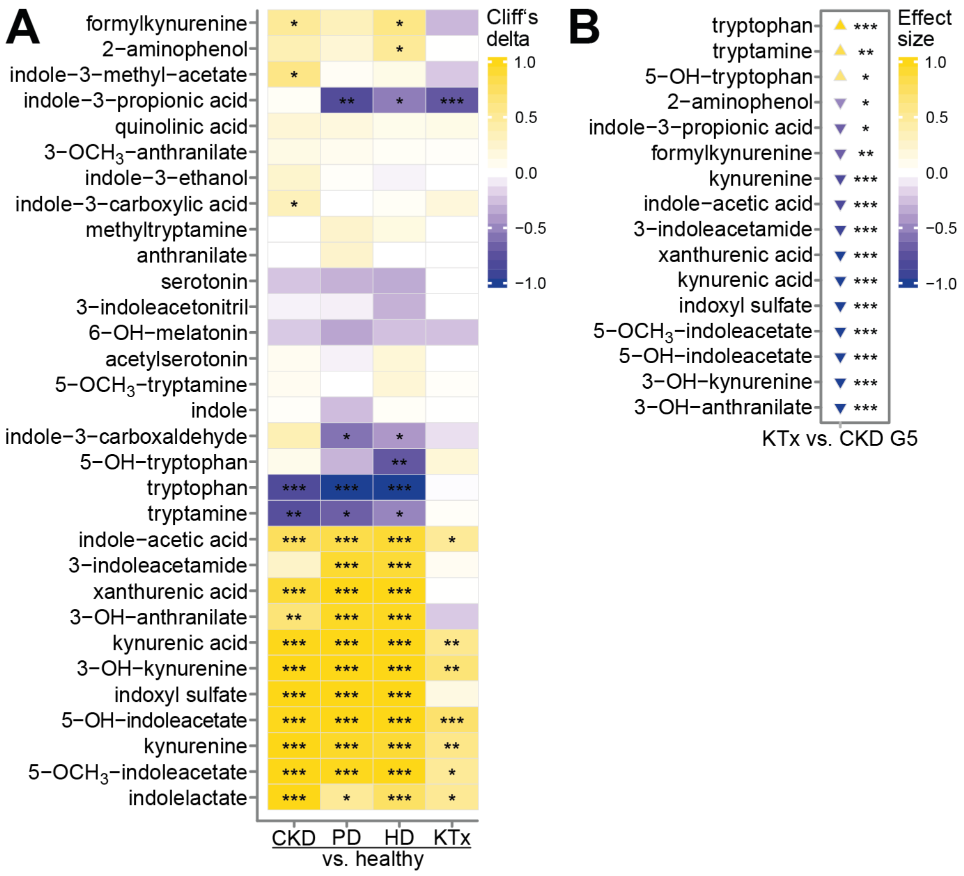

Figure S11. Individual tryptophan plasma metabolites II. Targeted plasma metabolomics was performed for 34 tryptophan metabolites, of which 31 could be quantified in the pediatric CKD cohort. **(A)** Heatmap showing group-to-group comparisons of individual tryptophan metabolites – in addition to highly abundant metabolites, several others were also dysregulated in CKD, including closely related tryptophan derivatives, which were decreased in CKD, and several metabolites of both the kynurenine and indole pathways, which were increased in CKD. **(B)** Cuneiform plot showing longitudinal changes in tryptophan metabolism after KTx with normalization of numerous metabolites. Melatonin, formylanthranilate and picolonic acid could not be quantified for technical reasons. **(A)** *$p < 0.1$, **$p < 0.01$, ***$p < 0.001$ by Wilcoxon rank-sum test with FDR correction. **(B)** *$p < 0.05$, **$p < 0.01$, ***$p < 0.001$ according to LongDat for FDR-corrected post-hoc test p-values. HD hemodialysis patients, KTx kidney transplant recipients, PD peritoneal dialysis patients.

Supplemental Figure 12

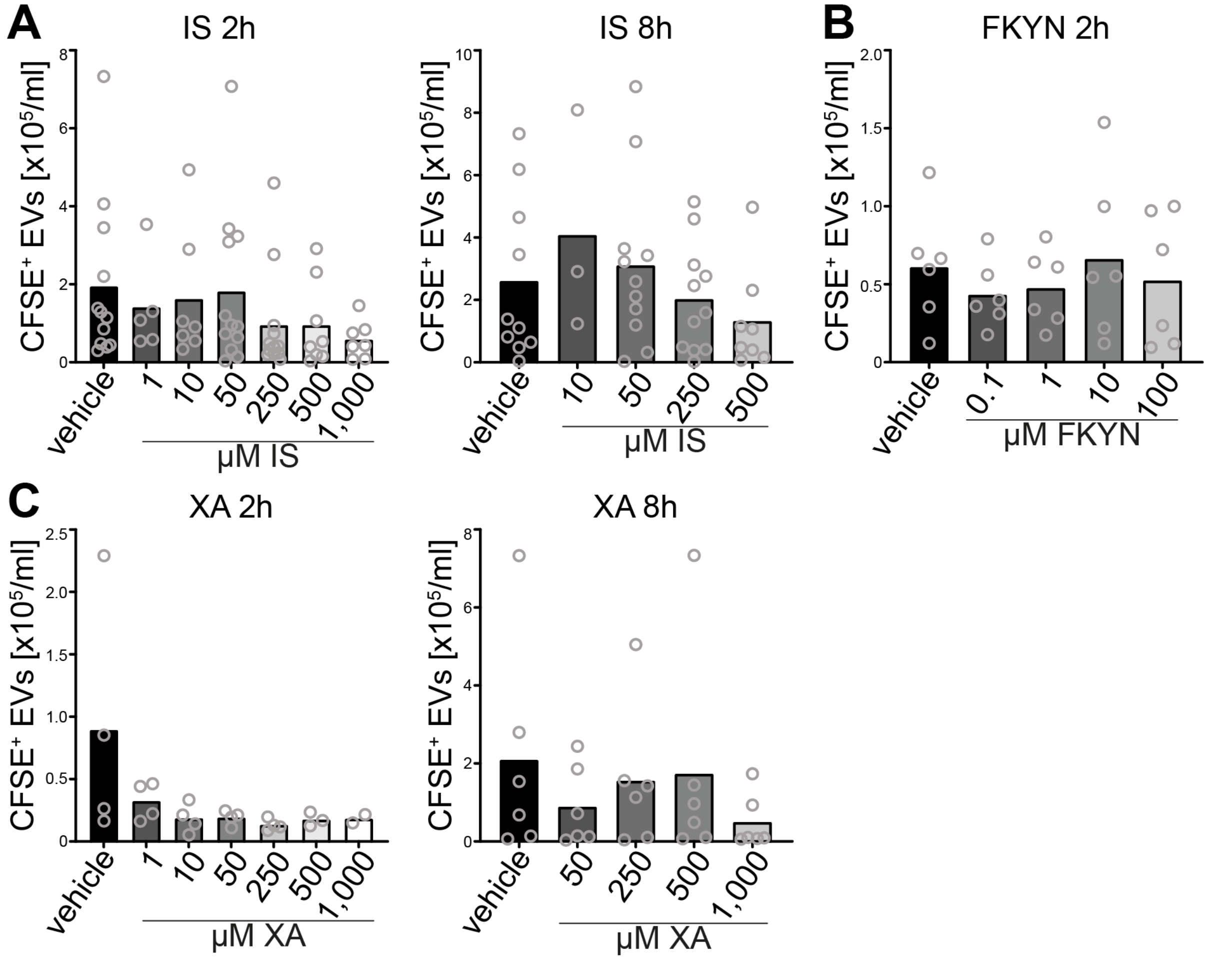

Figure S12. HAoEC EV release after exposure to uremic toxins. Quantification of CFSE^{+} membrane-stained Human Aortic Endothelial Cell (HAoEC) EVs after treatment for 2 or 8 hours with the uremic toxins **(A)** indoxyl sulfate (IS), **(B)** formylkynurenine (FKYN) or **(C)** xanthurenic acid (XA) with respective vehicle controls (DMSO for IS and XA, 0.6 mM NaOH for FKYN).

Supplemental Figure 13

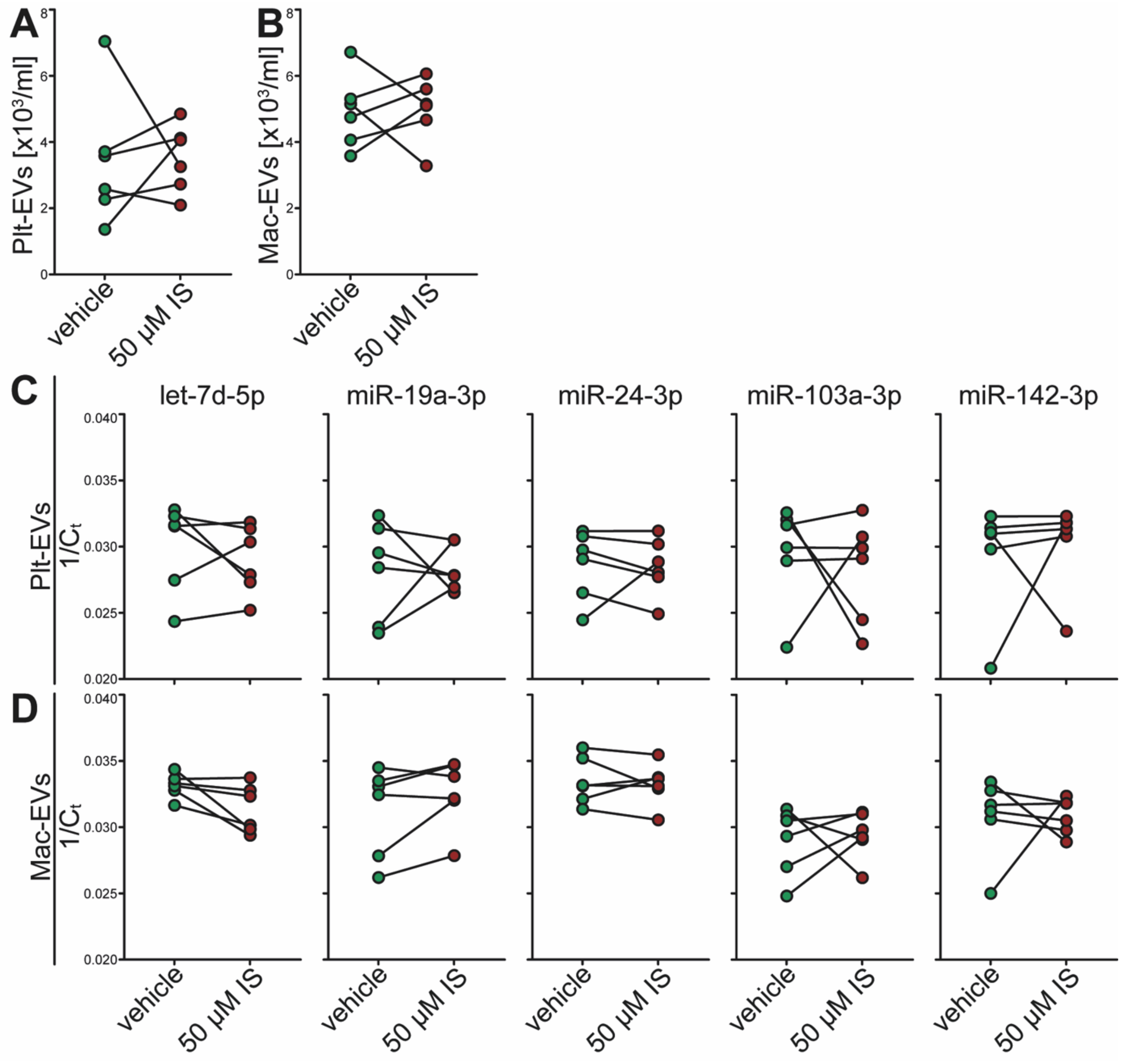

Figure S13. EV release from platelets and macrophages after exposure to indoxyl sulfate. (A,B) Flow cytometric quantification of (A) CD41^{+} EVs from human platelets treated with 50 µM indoxyl sulfate (IS) or vehicle (DMSO) for 3 hours and (B) CD68^{+} EVs from human monocyte-derived macrophages treated with 50 µM indoxyl sulfate (IS) or vehicle for 4 hours. (C,D) Plt- (C) and Mac-EV (D) miRNA cargo as determined by RT-qPCR. Each data point represents a single donor, lines connect IS and vehicle condition data from the same donors.

Supplemental Table 1

Component	Specifications
1.1 preanalytical variables	Blood was collected from pediatric CKD patients and age-matched healthy donors in 5 mL 3.2% sodium citrate (0.106 mmol/L) tubes (S-Monovette, Sarstedt, Nümbrecht, Germany) via brachial venipuncture using 21G or 23G cannulas. Before obtaining citrated blood for EV analyses, blood was drawn for routine clinical laboratory analyses. Cells were removed by an initial centrifugation at 600×g for 15 minutes, followed by two centrifugation steps at 2500×g for 15 minutes each to remove residual cells and debris. All centrifugation steps were performed without breaks. The platelet-free plasma was then snap frozen in liquid nitrogen and stored at -80°C.
1.2 experimental design	Aim: To compare the concentrations of circulating EVs of platelet, red blood cell, endothelial, B cell, T cell, monocyte, macrophage and neutrophil origin in CKD patients at different stages (before dialysis, on peritoneal dialysis, on hemodialysis, after kidney transplantation) with those in age-matched healthy donors.<br><br>Experimental variables: Platelet-free plasma was measured from 94 study participants in a cross-sectional study and 12 participants in a longitudinal follow-up after kidney transplantation was measured. Side scatter-based triggering was used for particle detection.
2.1 sample staining	Concentrations of EVs of platelet, red blood cell, endothelial, B cell, T cell, monocyte, macrophage and neutrophil origin were determined using antibody staining in 4 panels with two antibodies each:<br>- CD41 for platelets and CD235a for red blood cells<br>- CD14 for monocytes and CD31 for endothelial cells<br>- CD3 for T cells and CD20 for B cells<br>- CD66b for neutrophils and CD68 for macrophages<br><br>All panels included Annexin V staining as an internal control, which was not used for quantification. Details of reagents and concentrations are given in Table S2. 4 µL of platelet-free plasma was stained in a total volume of 200 µL, containing 50 µL CountBright Absolute Counting Beads (Thermo Fisher Scientific, Waltham, MA, USA), antibodies, Annexin V and Annexin V Binding Buffer (BioLegend, San Diego, CA, USA), for 15 minutes at room temperature and stored on ice until measurement. Antibodies, Annexin V and Annexin V Binding Buffer were filtered at 0.1 µm before use.
2.2 sample washing	No washing or dilution was performed prior to measurement. 0.1 µm filtration and centrifugation of the antibody ensured the specificity of the measured events for EVs.
2.3 sample dilution	4 µL platelet-free plasma was diluted 1:50 for staining and measured without further dilution.
3.1 buffer-only controls	A buffer-only control of 0.1 µm-filtered Annexin V Binding Buffer was acquired using the same acquisition settings as the biological samples, including trigger threshold, voltages and flow rate. The buffer-only control had an event rate of ~100 events/s.
3.2 buffer with reagent controls	Buffer with reagent controls of 0.1 µm-filtered Annexin V Binding Buffer, 0.1 µm-filtered Annexin V, counting beads and the corresponding 0.1 µm-filtered antibodies were recorded for each panel using the same acquisition settings as

	the biological samples, including trigger threshold, voltage and flow rate. The buffer with reagent controls had an event rate of ~100 events/s.
3.3 unstained controls	Unstained controls were measured at the same dilution as the matched stained, isotype and detergent controls. Acquisition settings were maintained for all samples, including trigger threshold, voltages and flow rate. The event rate of unstained controls differed by <10% from stained samples and isotype controls. No changes in scatter signal were observed between unstained controls, stained samples and isotype controls were observed. Fluorescence signals remained within a similar range in unstained and isotype controls.
3.4 isotype controls	Isotype controls were performed at the same antibody concentration as the stained samples and at the same sample dilution. Isotype controls were purchased from the same manufacturers as the respective antibodies. Acquisition settings were maintained for all samples, including trigger threshold, voltages and flow rate. No changes in scatter signal were observed between unstained controls, stained samples and isotype controls. Fluorescence signals remained within a similar range in unstained and isotype controls.
3.5 single stained controls	All antibodies and Annexin V were measured on single-stained samples. The compensation was determined based on the basis of the single-stained samples.
3.6 procedural controls	No washing, processing or purification was performed after staining. Therefore, no procedural controls were considered necessary.
3.7 serial dilutions	Serial dilutions of platelet-free plasma were performed in the range of 1:4 to 1:10,000. Dilutions of 1:25, 1:50 and 1:100 showed a linear decrease in event rate. Therefore, 1:50 was selected as the desired dilution for measurement of patient samples. Mean forward and side scatter, as well as fluorescence intensities were unchanged from 1:25 to 1:100.
3.8 detergent-treated controls	Detergent controls were performed on all panels. 20 µL of 1% 0.1 µm-filtered Triton X-100 was added to 200 µL of stained samples and incubated for 5 minutes at room temperature. Event rates decreased by ~50% and the percentage of positively stained events decreased by a further ~35%, indicating an overall binding of ~70%.
4.1 trigger channel and threshold	Detection was triggered on the 488 nm side scatter with a threshold of 0.39 arbitrary units based on the buffer only control and the unstained sample. The buffer only control had an event rate of ~100 events/s.
4.2 flow rate/volumetric quantification	Flow rate was kept constant across measured samples, as achieved by maintaining hydrodynamic settings and validated by the flow rate of calibration beads (Sphero Rainbow Calibration Particles (8 peaks), 3.0 - 3.4 µm, Becton Dickinson, Franklin Lakes, NJ, USA). Volumetric quantification was based on counting bead quantification and subsequent estimation of the measured volume. Calculated EV concentrations were then multiplied by 50 to account for a 1:50 plasma dilution.
4.3 fluorescence calibration	Photomultiplier voltages were set according to the staining intensity of positively stained EVs in single stained controls after setting antibody dilution adjustment and kept constant for all patient EV measurements. Fluorescence calibration with MESF or ABC beads was not performed.
4.4 scatter calibration	488 nm forward and side scatter detection voltages and side scatter threshold were set based on the buffer only control and the unstained sample. Scatter calibration based on Mie modelling was not performed.

5.1 EV diameter/surface area/volume approximation	Not performed.
5.2 EV refractive index approximation	Not performed.
5.3 epitope number approximation	Not performed.

Table S1. MiFlowCyt-EV guidelines report for plasma EV flow cytometry. Applicable to plasma EV flow cytometry in the pediatric CKD cohort and adult hemodialysis patients.

Supplemental Table 2

lineage	epitope	clone	isotype	fluorochrome	company	usage
platelet (Plt)	CD41	HIP8	mouse IgG1, κ	BV510	BioLegend	1:100
red blood cell (RBC)	CD235a	HI264	mouse IgG2a, κ	PerCP/Cy5.5	BioLegend	1:100
monocyte (Mo)	CD14	63D3	mouse IgG1, κ	BV421	BioLegend	1:100
endothelial cell (EC)	CD31	WM59	mouse IgG1, κ	BV711	BioLegend	1:100
T cell (T)	CD3	OKT3	mouse IgG2a, κ	BV421	BioLegend	1:100
B cell (B)	CD20	2H7	mouse IgG2b, κ	BV605	BioLegend	1:100
neutrophil (Neu)	CD66b	G10F5	mouse IgM, κ	BV421	BD	1:100
macrophage (Mac)	CD68	Y1/82A	mouse IgG2b, κ	BV785	BioLegend	1:400
Annexin V	Phosphatidylserin	-	-	FITC	BioLegend	1:4,000

Table S2. Plasma EV flow cytometry antibody list. Used in pediatric CKD cohort and adult hemodialysis patients.

Supplemental Table 3

metabolite	acquisition mode	precursor ion (m/z)	qualifier ion (m/z)	qualifier ion (m/z)	qualifier ion (m/z)	qualifier ion (m/z)	retention time (min)	quanti-fication
2-aminophenol	pos	110.060	92.040	65.110			2.1	yes
3-OH-anthranilate	neg	152.199	108.139	90.999	81.129		3.2	yes
3-OH-kynurenine	pos	225.210	207.820	162.380	110.200		2.3	yes
3-indoleacetamide	pos	175.086	130.065	103.055			3.8	yes
3-indoleacetonitril	pos	157.076	130.065	117.058			4.7	yes
3-OCH$_3$-anthranilate	pos	168.065	92.100	77.040			4.5	yes
5-OH-indoleacetate	pos	192.065	146.061	118.066			3.3	yes
5-OH-tryptophan	pos	221.092	204.066	162.055			2.6	yes
5-OCH$_3$-indoleacetate	pos	206.081	160.075	145.052	117.057		4.3	yes
5-OCH$_3$-tryptamine	pos	191.117	174.091	130.065			3.1	yes
6-OH-melatonin	pos	249.123	190.040	158.000			3.5	yes
acetylserotonin	pos	219.112	202.086	160.075			3.3	yes
anthranilate	pos	138.054	120.045	92.014	65.039		4.0	yes
formylkynurenine	pos	237.086	148.040	74.024			2.7	yes
indole	pos	118.065	91.100	65.100			5.3	yes
indole-3-carboxaldehyde	pos	146.060	117.040	91.040	89.040	65.100	4.3	yes
Indole-3-carboxylic acid	pos	162.054	144.000	118.058	116.058	88.986	4.4	yes
Indole-3-ethanol	pos	162.091	144.081	117.040	91.111		4.4	yes
Indole-3-methyl-acetate	pos	190.117	130.040	103.040	77.110		5.3	yes
Indole-3-propionic acid	pos	190.086	130.058	128.040	103.058		5.1	yes
Indole-acetic acid	pos	176.070	130.110	103.110	77.110		4.5	yes
indolelactate	pos	206.081	130.065	118.065			4.2	yes
Indoxyl sulfate	neg	212.002	132.370	80.160			3.5	yes
Kynurenic acid	pos	190.049	144.050	116.370	89.200		3.6	yes
kynurenine	pos	209.092	118.079	94.040			2.7	yes
methyltryptamine	pos	175.122	144.080	132.081			3.1	yes
quinolinic acid	pos	168.290	150.000	78.000			2.1	yes
serotonin	pos	177.102	160.076	132.045	115.033		2.5	yes
tryptamin	pos	161.107	144.081	117.070			3.1	yes
tryptophan	pos	205.097	118.200	115.040			3.3	yes
xanthurenic acid	pos	206.044	187.929	178.000	160.040	132.045	3.6	yes
formylanthranilate	pos	166.049	120.045	92.050			4.4	no
melatonin	pos	233.128	174.000	159.000			4.2	no
picolinic acid	pos	124.039	106.000	78.000			1.1	no

Table S3. List of tryptophan metabolites. Tryptophan metabolite transitions and feasibility of metabolite quantification after quality control assessment.

Supplemental Table 4

metabolite	Pearson *r*	*r* 95% CI	*p*
2-aminophenol	0.1947	-0.009411 to 0.3832	0.0615
3-OH-anthranilate	0.05611	-0.1493 to 0.2569	0.5932
3-OH-kynurenine	0.09001	-0.1158 to 0.2884	0.3909
3-indoleacetamide	0.1191	-0.08666 to 0.3152	0.2553
3-indoleacetonitril	-0.09953	-0.2972 to 0.1063	0.3425
3-OCH_3-anthranilate	0.02602	-0.1786 to 0.2285	0.8044
5-OH-indoleacetate	0.1053	-0.1006 to 0.3025	0.3151
5-OH-tryptophan	-0.1727	-0.3636 to 0.03214	0.0978
5-OCH_3-indoleacetate	0.1365	-0.06909 to 0.3310	0.1919
5-OCH_3-tryptamine	0.00006924	-0.2036 to 0.2038	0.9995
6-OH-melatonin	0.04108	-0.1640 to 0.2428	0.6959
acetylserotonin	0.04112	-0.1640 to 0.2428	0.6956
anthranilate	-0.04752	-0.2488 to 0.1577	0.6511
formylkynurenine	0.3404	0.1469 to 0.5088	**0.0008**
indole	-0.06455	-0.2648 to 0.1410	0.5388
indole-3-carboxaldehyde	0.01108	-0.1931 to 0.2143	0.916
Indole-3-carboxylic acid	-0.02897	-0.2313 to 0.1758	0.7828
Indole-3-ethanol	-0.09695	-0.2948 to 0.1089	0.3553
Indole-3-methyl-acetate	-0.02045	-0.2232 to 0.1840	0.8458
Indole-3-propionic acid	0.06491	-0.1407 to 0.2651	0.5365
Indole-acetic acid	0.07679	-0.1289 to 0.2762	0.4644
indolelactate	0.0469	-0.1583 to 0.2482	0.6553
Indoxyl sulfate	0.2223	0.01952 to 0.4076	**0.0322**
Kynurenic acid	0.08015	-0.1256 to 0.2793	0.445
kynurenine	0.06012	-0.1454 to 0.2606	0.567
methyltryptamine	-0.07138	-0.2711 to 0.1343	0.4966
quinolinic acid	0.02665	-0.1780 to 0.2291	0.7998
serotonin	0.04442	-0.1607 to 0.2459	0.6724
tryptamin	-0.09526	-0.2933 to 0.1106	0.3637
tryptophan	-0.2368	-0.4203 to -0.03480	**0.0223**
xanthurenic acid	0.2638	0.06349 to 0.4437	**0.0106**

Table S4. Tryptophan metabolite – endothelial EV correlation analyses. Individual plasma tryptophan metabolites were correlated with plasma endothelial EV concentrations in the pediatric CKD cohort including healthy donors and CKD patients. Pearson correlations were performed and $p < 0.05$ was considered statistically significant (bold). N=93 (one biospecimen missing in the healthy group).

Supplemental Table 5

	all	catheter	fistula
**individuals (N)**	16	7	9
**age (years)**	60.4 ± 4.2	48.6 ± 3.4	69.7 ± 5.1
**female**	5 (31%)	3 (43%)	2 (22%)

Table S5. Baseline clinical characteristics of the hemodialysis cohort. 16 hemodialysis patients with vascular access either via either a central venous catheter or an arterio-venous fistula were enrolled to analyze the immediate effects of dialysis.

References

1 Gulbins, A. *et al.* Antidepressants act by inducing autophagy controlled by sphingomyelin-ceramide. *Mol Psychiatry* **23**, 2324-2346, doi:10.1038/s41380-018-0090-9 (2018).

2 Gunther, A. *et al.* The acid ceramidase/ceramide axis controls parasitemia in Plasmodium yoelii-infected mice by regulating erythropoiesis. *Elife* **11**, doi:10.7554/eLife.77975 (2022).

3 Rogmann, J. J. Ordinal Dominance Statistics (Orddom): An R Project for Statistical Computing Package to Compute Ordinal, Nonparametric Alternatives to Mean Comparison (Version 3.1). *Available at: https://rdrr.io/cran/orddom/man/orddom-package.html* (2013).

4 Kassambara, A. & Mundt, F. Factoextra: Extract and Visualize the Results of Multivariate Data Analyses. R Package Version 1.0.7. *Available at: https://CRAN.R-project.org/package=factoextra* (2020).

5 Friedlander, M. R., Mackowiak, S. D., Li, N., Chen, W. & Rajewsky, N. miRDeep2 accurately identifies known and hundreds of novel microRNA genes in seven animal clades. *Nucleic Acids Res* **40**, 37-52, doi:10.1093/nar/gkr688 (2012).

6 Love, M. I., Huber, W. & Anders, S. Moderated estimation of fold change and dispersion for RNA-seq data with DESeq2. *Genome Biol* **15**, 550, doi:10.1186/s13059-014-0550-8 (2014).

7 Chen, C.-Y., Löber, U. & Forslund, S. K. LongDat: an R package for covariate-sensitive longitudinal analysis of high-dimensional data. *Bioinformatics Advances*, doi:10.1093/bioadv/vbad063 (2023).

8 Kuhn, M. Building Predictive Models in R Using the caret Package. *Journal of Statistical Software* **28**, 1 - 26, doi:10.18637/jss.v028.i05 (2008).

9 Wickham, R. *ggplot2: Elegant Graphics for Data Analysis. Available at: https://ggplot2.tidyverse.org/.* (Springer-Verlag New York, 2016).

10 Agarwal, V., Bell, G. W., Nam, J. W. & Bartel, D. P. Predicting effective microRNA target sites in mammalian mRNAs. *Elife* **4**, doi:10.7554/eLife.05005 (2015).

11 Mi, H., Muruganujan, A., Casagrande, J. T. & Thomas, P. D. Large-scale gene function analysis with the PANTHER classification system. *Nat Protoc* **8**, 1551-1566, doi:10.1038/nprot.2013.092 (2013).

12 Mi, H. *et al.* Protocol Update for large-scale genome and gene function analysis with the PANTHER classification system (v.14.0). *Nat Protoc* **14**, 703-721, doi:10.1038/s41596-019-0128-8 (2019).

13 Dobin, A. *et al.* STAR: ultrafast universal RNA-seq aligner. *Bioinformatics* **29**, 15-21, doi:10.1093/bioinformatics/bts635 (2013).

14 Liao, Y., Smyth, G. K. & Shi, W. featureCounts: an efficient general purpose program for assigning sequence reads to genomic features. *Bioinformatics* **30**, 923-930, doi:10.1093/bioinformatics/btt656 (2014).

15 Leek, J. T., Johnson, W. E., Parker, H. S., Jaffe, A. E. & Storey, J. D. The sva package for removing batch effects and other unwanted variation in high-throughput experiments. *Bioinformatics* **28**, 882-883, doi:10.1093/bioinformatics/bts034 (2012).

16 Kolde, R. pheatmap: pretty heatmaps version 1.0.12. *Avaialable at: https://github.com/raivokolde/pheatmap* (2019).

17 Gu, Z., Gu, L., Eils, R., Schlesner, M. & Brors, B. circlize Implements and enhances circular visualization in R. *Bioinformatics* **30**, 2811-2812, doi:10.1093/bioinformatics/btu393 (2014).

18 Maroski, J. *et al.* Shear stress increases endothelial hyaluronan synthase 2 and hyaluronan synthesis especially in regard to an atheroprotective flow profile. *Exp Physiol* **96**, 977-986, doi:10.1113/expphysiol.2010.056051 (2011).

19 Carpentier, G. *et al.* Angiogenesis Analyzer for ImageJ - A comparative morphometric analysis of "Endothelial Tube Formation Assay" and "Fibrin Bead Assay". *Sci Rep* **10**, 11568, doi:10.1038/s41598-020-67289-8 (2020).

20 Holle, J. *et al.* Inflammation in Children with CKD Linked to Gut Dysbiosis and Metabolite Imbalance. *J Am Soc Nephrol* **33**, 2259-2275, doi:10.1681/ASN.2022030378 (2022).
